# Identification of a Small Molecule Anti-biofilm Agent Against *Salmonella enterica*

**DOI:** 10.3389/fmicb.2018.02804

**Published:** 2018-11-20

**Authors:** Jasmine Moshiri, Darpan Kaur, Chido M. Hambira, Jenna L. Sandala, Jacob A. Koopman, James R. Fuchs, John S. Gunn

**Affiliations:** ^1^Department of Microbial Infection and Immunity, Infectious Diseases Institute, The Ohio State University, Columbus, OH, United States; ^2^Division of Medicinal Chemistry and Pharmacognosy, College of Pharmacy, The Ohio State University, Columbus, OH, United States

**Keywords:** biofilm, chronic infection, *Salmonella*, Typhoid, anti-biofilm

## Abstract

Biofilm formation is a common strategy utilized by bacterial pathogens to establish persistence in a host niche. *Salmonella enterica* serovar Typhi, the etiological agent of Typhoid fever, relies on biofilm formation in the gallbladder to chronically colonize asymptomatic carriers, allowing for transmission to uninfected individuals. *S. enterica* serovar Typhimurium utilizes biofilms to achieve persistence in human and animal hosts, an issue of both clinical and agricultural importance. Here, we identify a compound that selectively inhibits biofilm formation in both *S.* Typhi and *S.* Typhimurium serovars at early stages of biofilm development with an EC_50_ of 21.0 and 7.4 μM, respectively. We find that this compound, T315, also reduces biofilm formation in *Acinetobacter baumannii*, a nosocomial and opportunistic pathogen with rising antibiotic resistance. T315 treatment in conjunction with sub-MIC dosing of ciprofloxacin further reduces *S. enterica* biofilm formation, demonstrating the potential of such combination therapies for therapeutic development. Through synthesis of two biotin-labeled T315 probes and subsequent pull-down and proteomics analysis, we identified a T315 binding target: WrbA, a flavin mononucleotide-dependent NADH:quinone oxidoreductase. Using a *S.* Typhimurium strain lacking WrbA we demonstrate that this factor contributes to endogenous *S. enterica* biofilm formation processes and is required for full T315 anti-biofilm activity. We suggest WrbA as a promising target for further development of anti-biofilm agents in *Salmonella*, with potential for use against additional bacterial pathogens. The development of anti-biofilm therapeutics will be essential to combat chronic carriage of Typhoid fever and thus accomplish a meaningful reduction of global disease burden.

## Introduction

The resolution of chronic bacterial infections is often complicated by the inherent resistance of bacteria living within a biofilm community to antibiotic therapies. During a biofilm lifestyle, microorganisms secrete exopolysaccharides, DNA, and protein to form a protective extracellular matrix, allowing them to strongly adhere to surfaces and to other bacteria. The biofilm matrix acts as a physical barrier, shielding encased bacteria from immune and antibiotic perturbations (Jolivet-Gougeon and Bonnaure-Mallet, 2014; Van Acker et al., 2014; Watters et al., 2016). Additionally, entrance into a biofilm lifestyle introduces physiological changes allowing for emergence of persister cells within a bacterial population (Singh et al., 2009). The process of biofilm formation is characterized by sequential events that include initial attachment to a surface and secretion of matrix components followed by dispersal of community members to the environment (Costerton et al., 1995). One such bacterial infection which relies heavily on the formation of biofilms to achieve persistent infection (chronic carriage) and transmission to new hosts is that of *Salmonella enterica* serovar Typhi (*S.* Typhi), the causative agent of Typhoid fever. *S.* Typhi is of high clinical relevance in Southern Africa, South-central Asia, and South-eastern Asia, where contamination of food and water sources allows *S.* Typhi to spread to new hosts (Crump et al., 2004); in low- and middle-income countries, *S.* Typhi is estimated to have caused 11.9 million annual infections and 129,000 deaths in the year 2010 (Mogasale et al., 2014).

During an acute *S.* Typhi infection, ingested bacteria pass through the gastric barrier to reach the small intestine, where they cross the intestinal epithelium and are phagocytosed by macrophages to achieve systemic disease (Parry et al., 2002), reaching sites such as the ileum, liver, spleen, bone marrow, and gallbladder (Gonzalez-Escobedo et al., 2011). Following resolution of the acute infection, *S.* Typhi persists in the gallbladder of 2-5% of hosts through an asymptomatic chronic carrier state of disease (Merselis et al., 1964; Levine et al., 1982). During this chronic carriage state, *S.* Typhi forms biofilm communities on the surface of gallstones and invades the gallbladder epithelium (Crawford et al., 2010; Gonzalez-Escobedo et al., 2011). In a previous study, 88% of chronic carriers were observed to have gallstones, further demonstrating the role of gallstone biofilms in the mechanism of carriage (Schioler et al., 1983). Residence in the gallbladder results in intermittent shedding of *S.* Typhi into the feces of carriers (Crawford et al., 2010), providing a means for transmission to uninfected individuals.

The current standard of treatment for Typhoid carriage is treatment with fluoroquinolone antibiotics. A study completed in 1988 found that a 28-day course of norfloxacin achieved moderate success, resulting in resolution of infection in 86% of patients with normal gallbladders and 75% of individuals with gallstones (Gotuzzo et al., 1988), but the emergence of multi-drug resistant *Salmonella* strains in recent years presents a formidable challenge to this method of therapy (Pratap et al., 2012). The inherent resilience of biofilm-embedded bacteria presents an additional challenge for antibiotic therapy. Clinical *S. enterica* isolates growing within a biofilm can withstand antibiotic treatments that are lethal to planktonic cultures (Papavasileiou et al., 2010). In a murine model, the ability of *Salmonella* to aggregate on gallstone surfaces results in infections that are unable to be cleared by antibiotic therapy (Gonzalez et al., 2018). In patients with severe cholelithiasis, a cholecystectomy in conjunction with antibiotic therapy is often required to resolve the infection (Woo et al., 2001), although such invasive treatment for asymptomatic infection is not frequently available or undertaken in endemic regions.

As a human-restricted pathogen, *S.* Typhi depends on asymptomatic carriers as a reservoir for transmission. Given the importance of multicellular communities in the development of chronic carriage, the targeted treatment of *S. enterica* biofilm formation is an attractive strategy to impede the spread of *S.* Typhi via chronic carriage. When used in conjunction with antibiotic therapies, anti-biofilm agents have vast potential to reduce persistent infections within human populations (Kaplan, 2009). In this work, we identify a novel anti-biofilm agent against *Salmonella* and describe its potential for therapeutic use.

## Materials and Methods

### Bacterial Strains, Growth Conditions, and Compound Storage

Wildtype bacterial strains utilized in this study include *S.* Typhimurium ATCC 14028, *S.* Typhi Ty2, *Acinetobacter baumannii* (*A. baumannii*, ATCC 19606) and *Pseudomonas aeruginosa* PAO1 (*P. aeruginosa*). Overnight cultures were grown in Luria Bertani (LB) or Tryptic Soy Broth (TSB) at 37°C with aeration using a tissue culture rotator. The *S.* Typhimurium *ΔwrbA* mutant strain ^1^ (NR-42894) utilized was obtained from the McClelland collection of single-gene deletions (Porwollik et al., 2014) and contains a chloramphenicol resistance cassette in an ATCC 14028 background.

According to literature procedure (Lee et al., 2011), starting from 4-bromo acetophenone, T315 was synthesized in 6 linear steps as a white free-flowing powder (13% overall yield, MW 533.60 g/mol). T315 and related compounds were stored dry and not exposed to light. Stock solutions were prepared in dimethyl sulfoxide (DMSO) to a concentration of 5 mM and stored at -20°C. Drugs were administered via dilution in PBS or media as indicated, maintaining a final concentration of DMSO no greater than 5% (v/v).

### Biofilm Assay

The biofilm assays utilized here were adapted from literature procedure (O’Toole, 2011). A 24 h biofilm assay for *S.* Typhimurium included normalizing an overnight culture grown in LB to OD_600__nm_ = 0.8 (∼6.4 × 10^8^ cells/mL), diluting 1:100 into 1:20 TSB (diluted 1:20 into water) in a 96-well polystyrene flat-bottom microtiter plate (Corning, Product No. 3370), and incubating while nutating (using a Fisherbrand Variable Speed Nutator set at a 20° angle, 24 rpm) for 24 h at 30°C. Twenty four hour biofilm assays for *A. baumannii* and *P. aeruginosa* were identical with the exception that cultures were diluted 1:100 into LB and incubated in static conditions.

*Salmonella enterica* serovar Typhi requires the use of alternative *in vitro* conditions to yield robust biofilm formation, including rich media, extended incubation times, and surfaces amenable to bacterial attachment. For *S.* Typhi biofilm assays, overnight cultures were normalized to OD_490__nm_ = 0.65 (∼5.2 × 10^8^ cells/mL), diluted 1:6 into undiluted TSB, and incubated statically in a 6-well flat-bottom microplate (Corning, Product No. 3516) for 3 h at 37°C in 5% CO_2_. Following the 3 h incubation, bacteria were diluted 1:2500 into undiluted TSB, placed into a sterile polystyrene 96-well plate, and exposed to drug while nutating (20° angle, 24 rpm) for 96 h at 30°C, with removal of media and addition of fresh TSB and drug every 24 h. In *S.* Typhi biofilm assays, sterile polystyrene 96-well flat-bottomed plates had been initially coated with cholesterol via addition and subsequent evaporation (by overnight incubation at room temperature) of 100 μL per well of a 5 mg/mL cholesterol solution dissolved in equal proportions isopropanol/ethanol.

In these assays, drugs tested for anti-biofilm activity and appropriate vehicle controls were administered to wells concurrently with the addition of bacteria into 96-well polystyrene plates. After 24 h incubation (96 h incubation for *S.* Typhi), bacterial growth was determined by reading absorbance on a spectrophotometer (Molecular Devices, SpectraMax M5) at 600 nm. After washing plates vigorously by submerging in H_2_O to eliminate planktonic cells, biofilms were heat fixed (1 h, 60°C), stained with 33% crystal violet solution (6.0 mL PBS, 3.3 mL crystal violet, 333 μL methanol, 333 μL isopropanol) for 5 min, washed 3 times, and treated with 33% glacial acetic acid to release the stain. Biofilms were quantified by recording the optical density of the released dye (570 nm). All assays were performed at least three times, each in triplicate.

### Delayed Drug Addition Biofilm Assay

Delayed drug addition biofilm assays were conducted using the same bacterial growth conditions and crystal violet staining technique as utilized in *S.* Typhimurium 24 h biofilm assays, with the exception of delaying the addition of T315 or a DMSO control to developing biofilms. The addition of T315 to developing biofilms was delayed to 1, 3, and 6h after initiation of the 30°C incubation stage. Plates were incubated for 15 min on an orbital shaker at room temperature after each drug addition to ensure even drug dispersal throughout the wells. All assays were performed at least three times, each in triplicate.

### Viability Assay

To determine bactericidal or bacteriostatic properties, *S.* Typhimurium and *A. baumannii* were grown at 37°C in LB with aeration using a tissue culture rotator in the presence of 10 μM T315 or a vehicle control. Drug was administered via a 1:5 dilution of a 50 μM working solution of 1% DMSO in TSB (diluted 1:20). Samples taken over a 24 h timecourse were enumerated by serial dilution and drip plating onto LB agar (incubation for 16 h at 37°C).

### EC_50_ Determination

The half maximal effective concentration (EC_50_, defined as the concentration of drug which induces a response halfway between the baseline and maximum) was calculated using measurements of *S.* Typhimurium biofilm inhibition at concentrations of drug from 1 to 100 μM (24 h at 30°C) via the 24 h biofilm assay (previously described). Drug administration (at *t* = 0 h) was performed via 1:2 dilution of 2× stocks to yield desired drug concentrations and 5% DMSO in wells. Addition of DMSO to 5% in wells was included as control. Biofilms were quantified using the crystal violet stain and dye release technique.

### T315-Ciprofloxacin Combination Assays

In previous studies we have determined the minimum inhibitory concentrations (MIC) of our laboratory stocks of wildtype *S.* Typhimurium and *S.* Typhi to be 125 ng/mL and 500 ng/mL, respectively (Gonzalez et al., 2018). Ciprofloxacin (Sigma-Aldrich, Cat No. 17850) was stored at 10 mg/mL in H_2_O and administered by serial dilution in media (containing 20 μM T315 or DMSO, ensuring that previous T315 concentrations were maintained) to yield desired ciprofloxacin concentrations. Combination assays for *S.* Typhimurium and *S.* Typhi were prepared under the same conditions as Biofilm assays for the respective strains. As in previously described biofilm assays, T315 was administered concurrently with the addition of bacteria to wells, immediately prior to incubation at 30°C. After 6 h (*S.* Typhimurium; without removal of planktonic bacteria) or 24 h (*S.* Typhi; concurrently with the first instance of media replenishment) of incubation at 30°C, ciprofloxacin was added to wells at final concentrations ranging from 1 ng/mL to 10 μg/mL. After administration of ciprofloxacin, 30°C incubation was resumed. In *S.* Typhi assays, media was replaced with re-addition of appropriate drug concentrations every 24 h. After 24 h (*S.* Typhimurium) or 96 h (*S.* Typhi) of total incubation at 30°C, planktonic growth was measured via optical density readings (600 nm). Biofilms were quantified using the crystal violet stain and dye release technique. Assays were performed in biological triplicate.

### Isolation of Bacterial Lysate

*S. enterica* serovar Typhimurium was grown in LB at 37°C with aeration using a tissue culture rotator for 16 h, and 180 mL of bacterial culture was pelleted by centrifugation (10 min at 2464 ×*g*, followed by 10 min at 4500 ×*g*). Two consecutive centrifugation steps were applied to maximize pellet formation. Working at 4°C, bacterial pellets were resuspended in sonication buffer (50 mM Hepes, 120 mM NaCl, 20 mM MgCl_2_, pH 7.4, cOmplete^TM^ EDTA-free protease cocktail inhibitor [Roche]). The protein lysate was produced by sonication on ice twice for 1 min each and an additional 6 min with 10 s pulses at 50A. The bacterial lysate was cleared of cell debris via centrifugation (4500 ×*g*, 4°C, 20 min) and stored at -20°C for short-term use. Protein concentration (3.867 mg/mL) was quantified via bicinchoninic acid assay (Pierce BCA Protein Assay Kit)

### T315-Target Binding Identification

Chemical structures, synthesis routes, and purity analyses of T315-Biotin probes (biotinylated via a polyethylene glycol linker at two distinct sites, T315-S1 and T315-S2) can be found in the Supplementary Material. Briefly, alkyne-containing T315 derivatives were generated through a series of functional group transformations, and subsequently ‘clicked’ onto an azide-linked biotin tag under copper (I)-catalyzed cycloaddition conditions to produce two mechanistic probes, T315-S1 (0.52% overall yield, 11.3 mg, MW 1016.20 g/mol) as a clear residue, and T315-S2 (0.91% overall yield, 6.6 mg, MW 1002.17 g/mol) as an off-white film.

The Pierce^TM^ Pull-Down Biotinylated Protein:Protein Interaction Kit (Thermo) was utilized to identify T315-interacting proteins from a *S.* Typhimurium lysate. T315-Biotin probes were solubilized to 100 μM in DMSO. T315-Biotin probes were diluted ten-fold into 1:20 TSB to yield a 10 μM concentration; a No Drug control was prepared by diluting DMSO ten-fold into 1:20 TSB. After preparing columns with immobilized streptavidin, 400 μL of either 10 μM T315-S1, 10 μM T315-S2, or the No Drug control was loaded to a column and incubated at 23°C for 2.5 h, nutating (20° angle, 24 rpm). Columns were centrifuged at 1250 ×*g* for 1 min, and flow-through was collected; referred to as “Bait flow-through.” Biotin Blocking Solution was then added to columns as described by the kit manual and columns were washed twice using 1:20 TSB. Next, 475 μL of *S.* Typhimurium cleared lysate (3.867 mg/mL) was added to each column and incubated at 23°C for 3 h, nutating. Columns were centrifuged at 1250 ×*g* for 1 min, and flow-through was collected; referred to as “Lysate flow-through.” Columns were washed once with BupH^TM^ Tris-Buffered Saline (TBS). After addition of 10 μL neutralization buffer, column-bound proteins were eluted into 250 μL of Elution Buffer (Pierce^TM^, pH 2.8) and collected; referred to as “Elution 1.” Elution step was repeated once, and this sample is referred to as “Elution 2.”

Washes and eluent were examined by loading onto 10% SDS-PAGE, and gels were stained with Coomassie Brilliant Blue for primary visualization and imaging purposes or with SYPRO Ruby Protein Gel Stain (Life Technologies) for visualization and mass spectrometry analysis. The band of interest was excised from a SYPRO Ruby-stained polyacrylamide gel, digested and analyzed via liquid chromatography-tandem mass spectrometry at The Ohio State University Campus Chemical Instrument Center Mass Spectrometry and Proteomics Facility. Identified peptides were matched to the *S.* Typhimurium ATCC 14028 database (Accession: PRJNA33067) with the constraints of a 1% false discovery rate and identification of at least two unique peptide sequences.

### Data Analysis

Unless otherwise stated, all experiments are representative of at least 3 biological replicates. All data analysis was performed using GraphPad Prism 7, using statistical analyses as described for each independent experiment. Statistical significance was defined by a *P-*value less than 0.05.

## Results

### T315 Reduces Early Stages of *S.* Typhimurium Biofilm Formation

**FIGURE 1:**
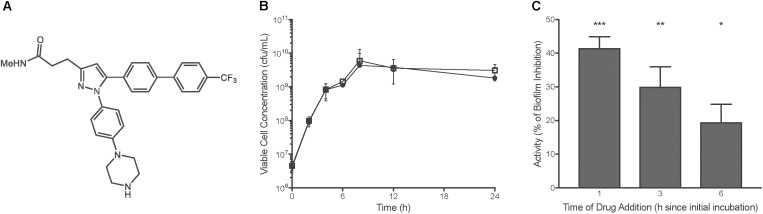
T315 inhibits early stages of *S.* Typhimurium biofilm formation. **(A)** Chemical structure of T315. **(B)** *S.* Typhimurium viability over 24 h is unchanged in the presence of 10 μM T315 (white squares) as compared to the DMSO control (black circles). Multiple *t*-tests with the Holm-Sidak correction for multiple comparisons revealed no significant differences at any timepoint. **(C)** In delayed drug administration assays, delivery of 5 μM T315 to developing biofilms was delayed 1, 3, and 6 h post-initial incubation. Average anti-biofilm activity of T315 against *S.* Typhimurium biofilms was calculated by comparisons of biofilm levels to a DMSO control. ^∗∗∗^*p* < 0.001, ^∗∗^*p* < 0.01, ^∗^*p* < 0.05; repeated measures one-way ANOVA.

We sought to identify new *Salmonella* biofilm inhibitors. A screen of 90 compounds originally developed as kinase inhibitors was initiated by adding the compounds to diluted bacteria in 96-well plates and examining biofilm formation after 24 h. In this screen, we fortunately identified T315 (Figure 1A), a compound that reduced formation of *S.* Typhimurium biofilms by 59.4% at the 5 μM concentration used in preliminary screening (results not shown). In previous work, prostate and mammary epithelial cells did not show cytotoxicity with T315 at concentrations between 1 and 5 μM (Lee et al., 2011), and the half-maximal lethal dose of T315 against normal T cell and B cell lymphocytes was greater than 10 μM (Liu et al., 2014). Evaluation of bacterial viability over a 24 h timecourse revealed no significant differences between *S*. Typhimurium planktonic growth in the presence of T315 as compared to a DMSO-treated control (Figure 1B). Therefore, T315 appears to limit biofilm formation specifically, rather than by exhibiting bacteriostatic or bactericidal activity.

To differentiate between inhibitory and dispersive anti-biofilm effects, T315 administration was delayed up to 6h post-initiation of the biofilm assay. Delayed administration of T315 results in a reduction in anti-biofilm activity (Figure 1C). At drug administration 6h post-initial incubation, 5 μM T315 exhibited only a 20% reduction in biofilm formation, as compared to 30% reduction at 3 h post-incubation, and 42% at 1h post-incubation. Thus, even a 1-h allowance for *Salmonella* to begin the biofilm formation processes prior to T315 exposure reduced the anti-biofilm activity of this compound. Dispersion assays delaying T315 administration by 24 h indicated that T315 is ineffective at dispersing a mature *Salmonella* biofilm (data not shown).

### T315 Exhibits a Half-Maximal Effective Concentration of 7.381 μM Against *S.* Typhimurium

**FIGURE 2:**
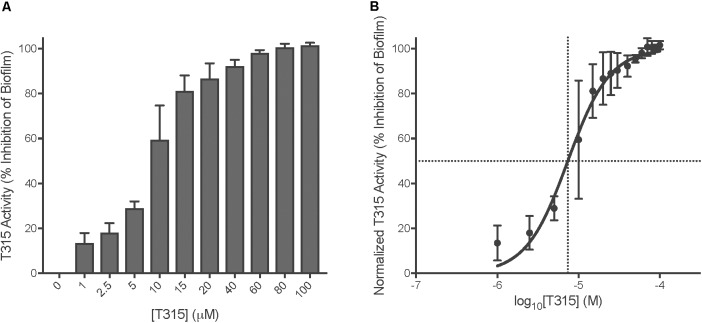
EC_50_ of T315 against *S.* Typhimurium biofilms is 7.4 μM. **(A)** Determination of the half-maximal effective concentration of T315 was achieved by exposing *S.* Typhimurium to drug concentrations from 1 to 100 μM and examining the levels of subsequent anti-biofilm activity after 24 h. **(B)** EC_50_ [7.4 μM (95% CI, 6.3–8.6 μM)] calculated using GraphPad Prism 7 to plot normalized T315 activity (percent of biofilm inhibition) as a function of log_10_ drug concentration and fitting of the dose-response curve (log[agonist] vs. normalized response, variable slope).

The half-maximal effective concentration (EC_50_) of T315 against *S.* Typhimurium biofilm formation was calculated by evaluating anti-biofilm activity, defined as percent inhibition of biofilm formation relative to an untreated control, at a range of concentrations between 1 and 100 μM (Figure 2). The EC_50_ was determined using Prism Graphpad 7 to normalize the data (such that the highest observed level of anti-biofilm inhibition achieved throughout the concentration range tested was stated as 100% normalized T315 activity, while activity of untreated controls was defined at 0%) and fit a dose-response curve. The EC_50_ was found to be equal to 7.4 μM with a 95% confidence interval from 6.3 to 8.6 μM. While this is higher than the 5 μM concentration utilized in the original screening, we consider it a more accurate description; we attribute this discrepancy to a change in drug stock origin from the preliminary screening.

### T315 Exhibits Anti-biofilm Properties Toward Gram-Negative Pathogen *A. baumannii*

**FIGURE 3:**
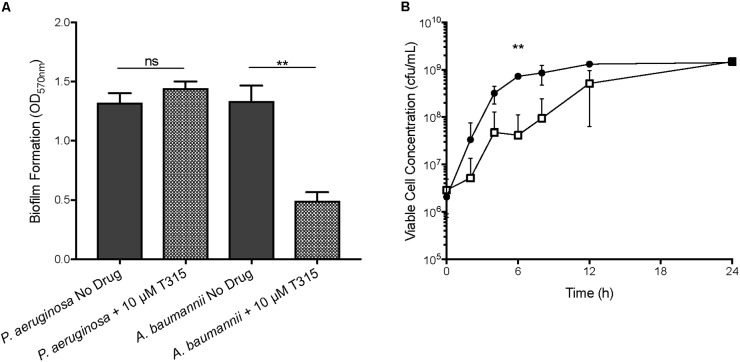
Evaluation of T315 anti-biofilm effects on other Gram-negative pathogens. **(A)** After 24 h exposure of 10 μM T315, *A. baumannii* biofilm formation was reduced by an average of 61.2%. Exposure of *P. aeruginosa* PAO1 to 10 μM T315 did not significantly reduce biofilm formation after 24 h. ^∗∗^*p* < 0.01; ratio paired *t*-test. **(B)** *A. baumannii* growth is significantly affected by 10 μM T315 (white squares) as compared to the DMSO control (black circles) at one intermediate point over a 24 h timecourse. ^∗∗^*p* < 0.01; multiple *t*-tests with the Holm-Sidak correction for multiple comparisons.

Anti-biofilm activity of T315 toward additional Gram-negative pathogenic biofilm forming species *A. baumannii* and *P. aeruginosa* was evaluated (Figure 3A). Twenty four hour biofilm assays indicate that at a concentration of 10 μM, T315 significantly reduces *A. baumannii* biofilm formation by an average of 61.2%. 10 μM T315 did not reduce *P. aeruginosa* PAO1 biofilm formation (Figure 3A), nor did addition of 20 μM T315 (data not shown).

Viability assays were used to determine the potential bacteriostatic or bactericidal effects of 10 μM T315 toward *A. baumannii*. Although decreased *A. baumannii* growth was observed upon T315 exposure at several points during the 24 h period, after 24 h of growth bacterial titers had recovered to levels similar to the DMSO-treated control (Figure 3B). This difference was statistically significant at the *t* = 6h timepoint; however, return of bacterial growth in T315-treated cultures after 24 h to levels similar to untreated suggests that the decrease in biofilm after 24 h is not a direct result of bactericidal activity.

### T315 Demonstrates Potential for Development as an Anti-typhoid Carrier State Therapeutic

**FIGURE 4:**
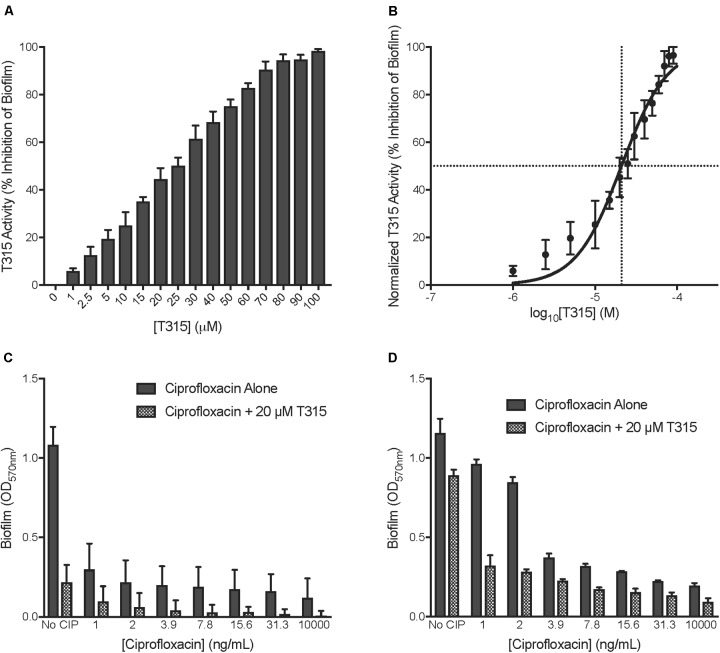
Potential for development as anti-Typhoid carrier therapeutic. **(A)** Determination of EC_50_ of T315 against *S.* Typhi biofilms was achieved by exposing *S.* Typhi to T315 concentrations from 1 to 100 μM and examining the levels of subsequent anti-biofilm activity after 96 h. **(B)** The EC_50_ of T315 against *S.* Typhi biofilms is 21.0 μM (95% CI, 18.9–23.3 μM). The EC_50_ was calculated using GraphPad Prism 7 to plot normalized T315 activity (percent of biofilm inhibition) as a function of log_10_ drug concentration and fitting of the dose-response curve (log[agonist] vs. normalized response, variable slope). **(C,D)** Combination of 20 μM T315 and sub-MIC ciprofloxacin (various concentrations) dosing demonstrates that T315 exhibits potential to be used in conjunction with low-dose antibiotics to effectively inhibit *Salmonella* biofilm formation. Sub-MIC doses of ciprofloxacin were added to developing **(C)** *S.* Typhimurium and **(D)** *S.* Typhi biofilms after 6 and 24 h of biofilm growth, respectively.

To simulate Typhoid chronic gallstone carriage *in vitro, S.* Typhi biofilms were grown for 96 h on cholesterol-coated polystyrene plates. In all experiments that have been performed to date, cholesterol-coating mimics experimental data observed with cholesterol gallstones from humans (Crawford et al., 2010). Upon T315 treatment, biofilm development was inhibited in a dose-dependent manner, without inhibition of planktonic growth (data not shown). Using cholesterol-coated assays, we observed that T315 is effective against *S.* Typhi, leading to a near-total (98.2%) reduction of biofilm at the highest tested concentration of 100 μM (Figure 4A). After normalization of the data and fitting a dose-response curve with Prism Graphpad 7, the EC_50_ of T315 against *S.* Typhi biofilm formation was determined to equal 21.0 μM, with a 95% confidence interval of 18.9–23.3 μM (Figure 4B).

Resolution of Typhoid carriage with antibiotic treatment alone is often complicated by both the emerging threat of antibiotic resistance globally as well as the inherent antibiotic resistance conferred by the biofilm lifestyle. Interestingly, recent work has demonstrated that sub-lethal antibiotic treatments can afford some protection against *S. enterica* clinical isolate biofilms (Majtan et al., 2008). Through a combination of low-dose antibiotic and T315 treatment, we observed strong reduction of *Salmonella* biofilms (Figures 4C,D). Sub-MIC concentrations of ciprofloxacin ranging from 1 to 31.3 ng/mL were included in our evaluation as well as a lethal ciprofloxacin dose of 10,000 ng/mL. While treatment of *S.* Typhimurium with 31.3 ng/mL ciprofloxacin reduced biofilm formation by 84.5%, addition of 20 μM T315 brought this to a total decrease in biofilm formation of 98.3% relative to untreated (Figure 4C). In *S.* Typhi, combination of 31.3 ng/mL ciprofloxacin with 20 μM T315 resulted in an average of 88.3% depletion in biofilm formation, as compared to 80.4 and 22.8% inhibition observed with either treatment alone, respectively (Figure 4D). Altogether, these results demonstrate that T315 can be used in conjunction with currently available antibiotic therapies to enhance the prevention of biofilm formation in the clinically relevant *S. enterica* serovars of *S.* Typhi and *S.* Typhimurium.

### T315 Targets WrbA to Inhibit Biofilm Formation

To identify the target of T315 responsible for its anti-biofilm activity, we pursued a direct pull-down approach. T315 was synthesized with the addition of a biotin marker connected via a polyethylene glycol linker at either the terminal end of the piperazine ring (S1) or the amide moiety (S2), yielding two probes: T315-S1 and T315-S2. Synthesis of T315-Biotin probes is detailed in the Supplemental Methods and Supplementary Figure S1A.

Utilizing both T315-Biotin probes, we performed a pull down of T315-interacting molecules from *S.* Typhimurium stationary-phase cellular lysates. We analyzed the flow-through and eluent that resulted from the pull down with T315-S1, T315-S2, and a no drug (DMSO-treated) control by SDS-PAGE (Supplementary Figure S1B). We observed one band near 20 kDa in size present in the eluent from both T315-Biotin probes that was not present in the no drug control. This band of interest in the T315-S1 Eluent 1 lane was excised, digested and analyzed via liquid chromatography-tandem mass spectrometry (LC-MS/MS). Other minor bands were visible in the stained SDS-PAGE but not pursued at this time.

**FIGURE 5:**
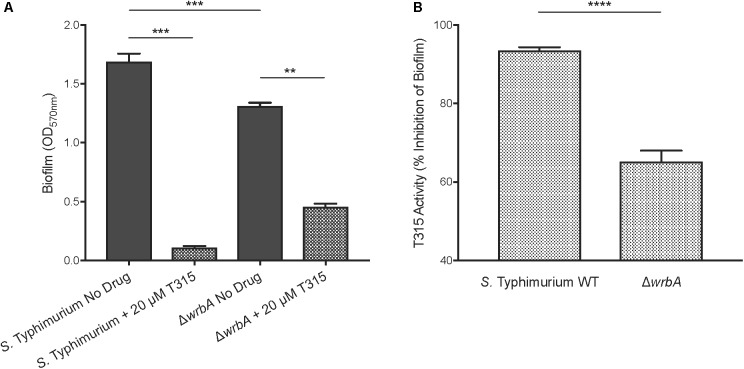
T315 targets WrbA to inhibit *S.* Typhimurium biofilm formation. **(A)** In the absence of drug treatments, *S.* Typhimurium *ΔwrbA* establishes reduced biofilm formation, 22.4% on average, relative to wildtype (^∗∗∗^*p* < 0.001; unpaired *t*-test). The addition of 20 μM T315 reduced biofilm formation in both *S.* Typhimurium wildtype and Δ*wrbA* strains (^∗∗^*p* < 0.01, ^∗∗∗^*p* < 0.001; ratio paired *t*-test), though its anti-biofilm activity is weakened by loss of WrbA. **(B)** Transformed data clearly show that T315 anti-biofilm activity with 20 μM treatment was significantly reduced in Δ*wrbA* compared to wildtype, by an average of 30.3%, indicating a reliance on WrbA to achieve maximum biofilm inhibition (^∗∗∗∗^*p* < 0.0001; unpaired *t*-test).

Our mass spectrometry analysis indicated that the most abundant protein present in this band was the flavoprotein WrbA. We used a *S.* Typhimurium *ΔwrbA* mutant strain to evaluate the role that WrbA plays in T315 activity. Loss of WrbA in *S.* Typhimurium resulted in decreased ability to develop biofilms. In the absence of drug treatment, the *ΔwrbA* mutant was impaired in biofilm formation, achieving an average of 22.4% less biofilm compared to wildtype (Figure 5A). This does not appear to be due to a growth defect, as the overall bacterial growth of the *S.* Typhimurium *ΔwrbA* mutant was equal to or greater than wildtype levels, as measured by viability assay over a 24 h timecourse (Supplementary Figure S2).

Using our *S.* Typhimurium Δ*wrbA* strain, we evaluated T315’s dependence on the presence of WrbA to inhibit biofilm formation. 20 μM T315 was capable of significantly reducing biofilm formation in *S.* Typhimurium *ΔwrbA*, with an observed average biofilm inhibition of 65.2% (Figure 5). However, the level biofilm inhibition achieved by T315 against the *ΔwrbA* strain was significantly lower than those achieved against wildtype *S.* Typhimurium cultures (Figure 5B). On average, the anti-biofilm activity of 20 μM T315 was 30.3% lower in the absence of WrbA (*p* < 0.0001). Altogether, these results indicate that WrbA contributes to T315’s anti-biofilm activity, and it is likely one of the targets of T315 in *S.* Typhimurium.

## Discussion

*Salmonella enterica* serovar Typhi chronic gallbladder carriage is central to the spread of Typhoid fever throughout endemic regions. An effective strategy to decrease or eliminate the incidence of this disease could arise through a combination of anti-biofilm and bactericidal therapies to reduce chronic gallbladder carriage. In this work, we performed a screen of kinase inhibitor derivative compounds that identified a small molecule, T315, that specifically inhibits *S.* Typhimurium biofilm formation. T315 is effective at inhibiting early stages of *S.* Typhimurium biofilm formation without affecting bacterial viability. T315 has previously been identified as an integrin-linked kinase inhibitor and potential therapeutic against chronic lymphocytic leukemia (Lee et al., 2011). Previous studies have observed relatively low cytotoxic effects against non-cancerous human cell lines in the concentration range utilized in our work (Lee et al., 2011; Liu et al., 2014). T315 exhibits similar anti-biofilm activity against an additional Gram-negative biofilm-forming pathogen, *A. baumannii*, but is not active against *P. aeruginosa*, suggesting a level of specificity. The activity shown against *A. baumannii* is clinically relevant, as this is a member of the ESKAPE pathogens in dire need of new treatment methods, reviewed in Pendleton et al. (2013). Importantly, we have demonstrated that T315 can effectively impede *S.* Typhi biofilm formation, with an EC_50_ of 21.0 μM on cholesterol-coated surfaces *in vitro* that partially mimic the *in vivo* gallbladder environment mediating carriage.

Considering the development of anti-biofilm therapeutics, combination therapies could be an effective strategy to maximize efficiency and impede the development of resistance. We are interested in exploring the potential of combination therapies of anti-biofilm agents with sub-MIC dosing of traditional antibiotics. At sub-MIC doses, antibiotic exposure can significantly impact global gene regulation as well as the regulation of virulence and biofilm-associated factors (Matsui et al., 2005; Yim et al., 2006). By utilizing a combination of T315 and sub-MIC ciprofloxacin, we achieved near-total depletion (98.3%) of *S.* Typhimurium biofilms. *S.* Typhi biofilms were also strongly affected by the combination therapy, with an 88.3% reduction achieved; notably, this inhibition is significantly greater than the 80% depletion observed after treatment with a 10 μg/mL dose of ciprofloxacin, which is lethal to planktonic cells (*p* = 0.0038). These results demonstrate that T315-antibiotic combination therapies are an effective strategy to drastically reduce *Salmonella* biofilm formation *in vitro*. Future studies will be needed to evaluate optimal drug combinations and dosing regimens to move toward the development of therapies to target *in vivo* chronic gallbladder carriage.

To identify the target of T315 in *Salmonella*, we synthesized two biotin-labeled T315 probes and utilized these probes to perform a direct pulldown of T315-interacting proteins from *S.* Typhimurium cell lysates. A band of interest near 20 kDa was observed in eluent from both T315-Biotin probes used, but not in our negative control. Under constraints of a 1% false discovery rate and identification of at least two unique peptide sequences, our mass spectrometry analysis identified WrbA as the protein in this band. The role of WrbA in the mechanism of action of T315 was verified by comparing T315 anti-biofilm activity in wildtype and *ΔwrbA S.* Typhimurium, where T315 activity was reduced, presumably due to the loss of a target. It is important to note that while WrbA is required for full anti-biofilm activity, our data suggest that T315 activity is mediated by at least one additional, as yet unidentified, mechanism in *S.* Typhimurium.

WrbA is a 20.8 kDa protein with flavin mononucleotide (FMN)-dependent NADH:quinone oxidoreductase activity. In *Escherichia coli* (strain JM101), WrbA was originally described as directly binding to the tryptophan repressor TrpR-operator DNA complex and was subsequently named for this activity [tryptophan (*W*) *r*epressor-*b*inding protein] (Yang et al., 1993). However, additional work has shown no direct physical interaction between WrbA and the tryptophan repressor complex, but this interaction remains controversial (Grandori et al., 1998). The *E. coli* WrbA has 94% amino acid sequence identity to the *S.* Typhimurium 14028 homolog (NCBI Protein BLAST). *In vitro*, WrbA demonstrates NAD(P)H:quinone redox activity (Patridge and Ferry, 2006), likely binding NADH and quinone substrates in a mutually exclusive fashion and functioning through a ping-pong kinetic mechanism (Kishko et al., 2012; Degtjarik et al., 2016). A 2014 study demonstrated that WrbA contributes to *P. aeruginosa* antioxidant defense, proposing that WrbA aids the oxidative stress response in low H_2_O_2_ conditions (Green et al., 2014).

WrbA has previously been implicated in the biofilm lifestyle of Gram-negative bacteria, including *Salmonella* species. Mangalappalli-Illathu and Korber (2006) continually exposed *S. enterica* serovar Enteritidis biofilms to prolonged sub-lethal doses of the cationic surfactant benzalkonium chloride. In biofilms that developed adaptive resistance, the expression of WrbA—as well as other antioxidant factors including superoxide dismutase SodB, thiol peroxidase Tpx, and putative peroxidase STY0440—were significantly upregulated (Mangalappalli-Illathu and Korber, 2006). In *E. coli*, sub-MIC doses of zosteric acid are capable of significantly reducing biofilm formation; using a direct pulldown and mass spectrometry approach, zosteric acid and derivative compounds were found to directly bind to WrbA (Catto et al., 2015). In a murine model of infection, deletion of WrbA hinders the ability of *Yersinia pseudotuberculosis* to establish a persistent infection (Avican et al., 2015).

WrbA expression is under the control of the general stress sigma factor RpoS (σ^38^) (Lacour and Landini, 2004; Lelong et al., 2007), which is induced during the nutrient-limited conditions of stationary phase. In addition to causing global cellular changes, RpoS induction stimulates upregulation of the key biofilm regulator CsgD, the expression of which leads to biosynthesis of extracellular matrix components such as curli fibers and cellulose (Prigent-Combaret et al., 2001; Brombacher et al., 2003; Ogasawara et al., 2011). Upon *csgD* deletion, non-aggregative *S.* Typhimurium was noted to downregulate WrbA expression relative to wildtype (White et al., 2010). Through chromatin immunoprecipitation and microarray analysis, the *wrbA-ymdF* promoter region was identified as one of 20 CsgD genomic binding sites in *E. coli* (Ogasawara et al., 2011), indicating direct regulatory control of WrbA expression by CsgD.

It is possible that WrbA plays a regulatory role in mediating the switch from virulence to a persistent, biofilm-forming lifestyle. WrbA was identified as one of several putative targets for a class of anti-virulence compounds against type III secretion in *E. coli* (Wang et al., 2011). While deletion of WrbA did not affect virulence against macrophages *in vitro*, transcriptional analysis by microarray during stationary phase growth provides clues toward understanding the regulatory networks of this protein. Among the 127 differentially regulated factors, genes involved in type II and type III secretion and virulence factors including hemolysin were significantly upregulated upon *wrbA* deletion, while those involved in flagellar biosynthesis including *flgB, flgC, fliA*, and *fliC* were significantly downregulated compared to wildtype (Wang et al., 2011). These observations are consistent with our finding that *S.* Typhimurium *ΔwrbA* is deficient in biofilm formation, achieving an average of 22.4% less biofilm formation than wildtype cultures. Altogether, these data lead us to speculate that inhibition of WrbA results in a reduced capacity to efficiently transition to a biofilm-forming lifestyle.

We can also speculate that the oxidoreductase activity of WrbA could be responsible for its role in biofilm formation. Through reaction with single-electron acceptors such as O_2_, reduced quinones are capable of generating reactive oxygen species (ROS). Oxidative stress by ROS can contribute to DNA, lipid, and protein damage, and can result in cell death (Imlay, 2013). It is possible that functional WrbA oxidoreductase activity might be required to prevent ROS accumulation in the cell, the buildup of which can affect biofilm formation (Gambino and Cappitelli, 2016). Alternatively, WrbA may utilize its FMN cofactor to function as a redox sensor, relaying the signal downstream to induce changes in cellular activities such as biofilm formation. Bacteria sense oxidative stress, ROS, and O_2_ using a variety of sensor molecules, and several examples of FMN cofactor-based redox sensors have been identified in bacteria (Rebbapragada et al., 1997; Green and Paget, 2004; Martinez-Argudo et al., 2004; Petrova and Sauer, 2012). Future work to define the relationship between WrbA and biofilm development will increase our understanding of this process and may identify novel targets for drug development.

The data presented here demonstrate that treatment with small molecules, such as T315, hold promise for development as anti-biofilm therapeutics. As we struggle to maintain effective antimicrobial therapies with the global threat of emerging resistance, novel strategies to thwart pathogens will need to be thoroughly explored. By targeting biofilm formation processes in *Salmonella* spp., we can help alleviate the spread of typhoid fever by chronically infected individuals, diminishing reservoirs of disease in patients who are unresponsive to antibiotic therapy.

## Author Contributions

JM designed and performed the experiments, analyzed the data, obtained funding, and prepared the manuscript. DK, JS, and JK designed and performed the experiments. DK and JS also reviewed the manuscript and contributed to the materials and methods. CH synthesized T315 and T315-Biotin chemical probes, performed purity analysis of synthesized compounds, and prepared the accompanying Supplemental Information. JF and JG oversaw and directed the research, obtained funding, and reviewed the manuscript.

## Conflict of Interest Statement

The authors declare that the research was conducted in the absence of any commercial or financial relationships that could be construed as a potential conflict of interest.

## Footnotes

1 beiresources.org
