# Supplementary material for: Identification of a Small Molecule Anti-biofilm Agent Against Salmonella enterica
Source: Front Microbiol. 2018 Nov 20;9:2804. doi: 10.3389/fmicb.2018.02804 (PMC6256085; doi:10.3389/fmicb.2018.02804)

frontiers

Supplementary Material

Identification of a Small Molecule Anti-Biofilm Agent Against *Salmonella enterica*

Jasmine Moshiri, Darpan Kaur, Chido M. Hambira, Jenna L. Sandala, Jacob A. Koopman, James R. Fuchs, John S. Gunn*

*** Correspondence:** Dr. John S. Gunn: john.gunn@osumc.edu

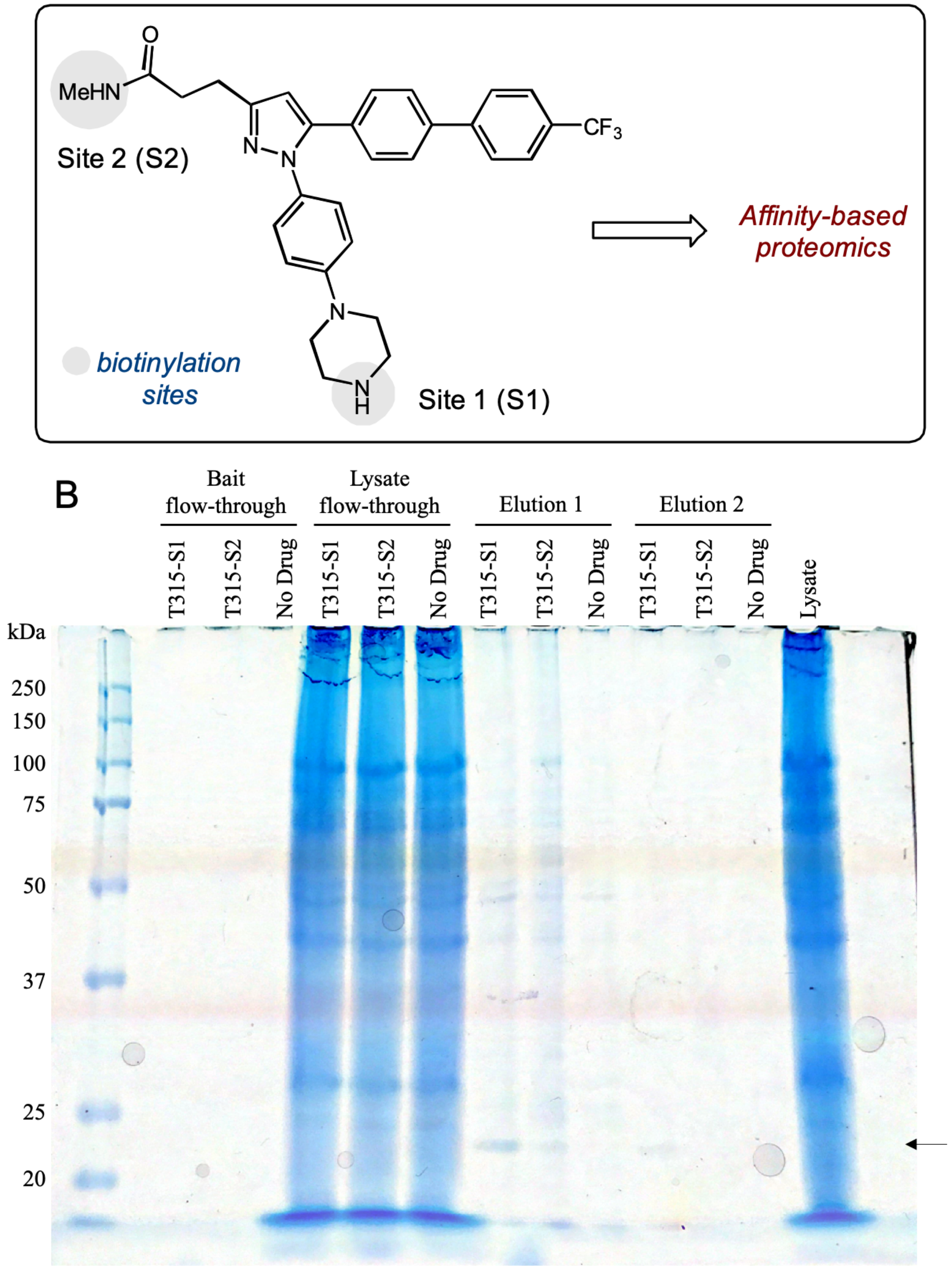

Figure S1. (A) Biotin connected via a polyethylene glycol linker was introduced to T315 at site 1 and at site 2 to yield T315-S1 and T315-S2 probes, respectively. (B) T315-Interacting protein pulldown was analyzed by SDS-PAGE. T315-S1 and T315-S2, or a no drug control were loaded onto an immobilized streptavidin column. Interacting proteins from *S.* Typhimurium soluble lysate were allowed to bind to the columns and subsequently eluted. Flow-through from the probe (bait) loading and cellular lysate steps, as well as two eluent steps were analyzed by 10% polyacrylamide gel electrophoresis, stained with Coomassie Brilliant Blue (above). T315-S1 Elution 1 band of interest (arrow) was excised from a replicate 10% polyacrylamide gel stained with SYPRO Ruby (not shown) for LC-MS/MS analysis. Image background subtracted, contrast adjusted using ImageJ. Precision Plus Protein™ All Blue Prestained Protein Standards (Bio-Rad) were utilized.

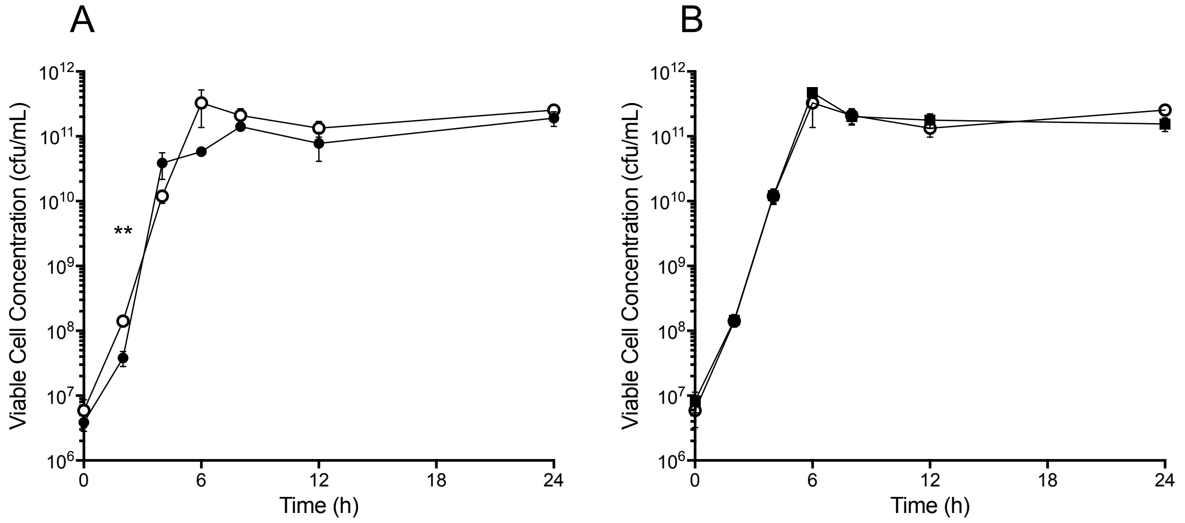

Figure S2. Examination of *S.* Typhimurium Δ*wrbA* bacterial growth. (A) We evaluated a timecourse of *S.* Typhimurium Δ*wrbA* growth (white circles) as compared to wildtype (black circles), observing significantly higher bacterial growth in Δ*wrbA* cultures at T=2h. (B) Treatment with 10 µM T315 (black squares) did not significantly alter *S.* Typhimrium Δ*wrbA* bacterial growth at any timepoint over 24h. **p<0.01; multiple t-tests with the Holm-Sidak correction for multiple comparisons.

Supplemental Methods: Synthesis and Analysis of T315-Biotin Probes

General Information

All reactions were carried out under an argon atmosphere with dry solvents under anhydrous conditions unless otherwise stated. Oven-dried syringes were used to transfer air and moisture sensitive liquids. All commercial reagents, anhydrous solvents, and reagent-grade solvents were purchased from Sigma-Aldrich, Fisher Scientific, VWR, or PurePEG and used as received without further purification, unless otherwise stated. Reactions were monitored by thin layer chromatography (TLC) using aluminum backed pre-coated silica gel plates from (TLC Silica Gel F-254, 200 µm, Dynamic Adsorbents) using UV light as the visualizing agent. Flash chromatography was performed using silica gel (60Å, pore size 32-63 µm, Dynamic Adsorbents). Silica gel was deactivated by first washing with a 10% triethylamine solution in the eluent and then washing with the eluent itself (3x) unless otherwise stated. Deuterated solvents for NMR were purchased from Cambridge Isotope Labs and used as received. NMR spectra were recorded on Bruker DPX250, AV300, or DRX400 MHz spectrometers and calibrated using the residual undeuterated solvent peak ($CDCl_3$: δ 7.26 ppm ^{1}H NMR, 77.16 ppm ^{13}C NMR; acetone-d_6: δ 2.05 ppm ^{1}H NMR, 206.26 ppm ^{13}C NMR; DMSO-d_6: δ 2.50 ppm ^{1}H NMR, 39.52 ppm ^{13}C NMR; CD_3OD: δ 3.31 ppm ^{1}H NMR, 49.00 ppm ^{13}C NMR). Proton (^{1}H) NMR data is reported as follows: chemical shift in ppm (multiplicity [as: s = singlet, d = doublet, t = triplet, q = quartet, quint = quintet, sext = sextet, sept = septet, m = multiplet, br = broad], coupling constant(s) in Hz, relative integration). Carbon (^{13}C) NMR data was reported as chemical shift (δ) in ppm. High resolution mass spectra (HRMS) were recorded on Thermo LTQ Orbitrap by electrospray ionization (ESI) time of flight experiments and reported as *m/z*.

Part I: Synthesis of Azide biotin linker

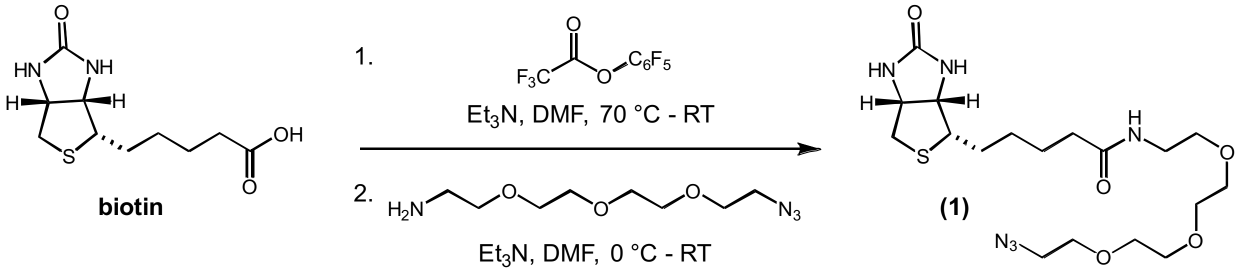

N-(2-(2-(2-(2-azidoethoxy)ethoxy)ethoxy)ethyl)-5-((3aS,4S,6aR)-2-oxohexahydro-1H-thieno[3,4-d]imidazol-4-yl)pentanamide. The azide biotin linker was prepared according to the literature procedure (Chambers et al. 2013). A solution of biotin (250 mg, 1.02 mmol) in DMF (5 mL) was heated to 70ºC for 10 minutes and allowed to cool to room temperature. Triethylamine (181.2 mg, 1.79 mmol) was added followed by pentafluorophenyl trifluoroacetate (405.60 mg, 1.45 mmol). The reaction was left to stir at room temperature for 30 minutes at which point it became pink. The reaction was concentrated under vacuum and the resulting residue was triturated with diethyl ether (10 mL) to afford biotin-pFp ester (382 mg, 0.931 mmol) as a white solid that was immediately carried forward without further purification. To a flask containing 11-azido-3,6,9-trioxaundecan-1-amine (203.5 mg, 1.02 mmol) and triethylamine (206.4 mg, 2.04 mmol) in DMF (13.3 mL) cooled to 0ºC was added a cooled solution of biotin-pFp ester in DMF (9 mL) dropwise. The reaction was left to stir at room temperature for 1 hour, then concentrated under vacuum. The resulting residue was triturated with diethyl ether (10 mL x 2) to afford **azide biotin linker**, **1** (275 mg, 0.62 mmol, 66%) as a white solid that was carried forward without further purification. ^{1}H NMR (400 MHz, MeOD) δ 4.49 (dd, J = 7.9, 4.3 Hz, 1H), 4.31 (dd, J = 7.9, 4.5 Hz, 1H), 3.70 – 3.64 (m, 8H), 3.64 – 3.60 (m, 2H), 3.55 (t, J = 5.5 Hz, 2H), 3.40 – 3.34 (m, 4H), 3.24 – 3.18 (m, 1H), 2.93 (dd, J = 12.7, 5.0 Hz, 1H), 2.71 (d, J = 12.7 Hz, 1H), 2.22 (t, J = 7.4 Hz, 2H), 1.79 – 1.55 (m, 4H), 1.45 (p, J = 7.4 Hz, 2H). ^{13}C NMR (101 MHz, MeOD) δ 176.14, 166.11, 71.67, 71.63, 71.53, 71.27, 71.13, 70.60, 63.37, 61.63, 56.99, 51.79, 41.04, 40.37, 36.73, 29.75, 29.50, 26.84. HRMS m/z calc'd for $C_{18}H_{32}N_6NaO_5S$ $[M+Na]^+$: 467.2047, found 467.2040.

Part II: Synthesis of T315-S1 Probe

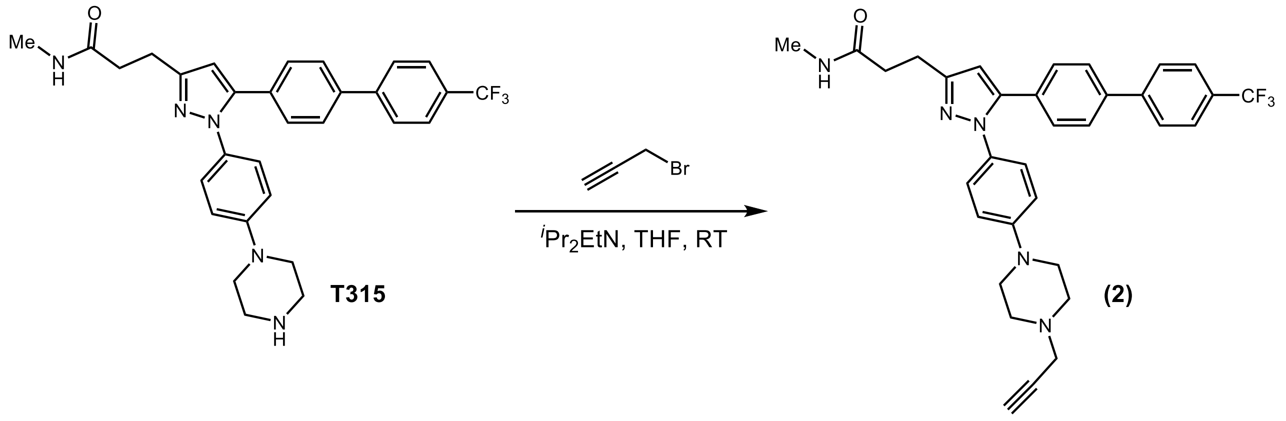

N-methyl-3-(1-(4-(4-(prop-2-yn-1-yl)piperazin-1-yl)phenyl)-5-(4'-(trifluoromethyl)-[1,1'-biphenyl]-4-yl)-1H-pyrazol-3-yl)propanamide. Synthesis of T315-S1 Probe began from a known intermediate OSU-T315 prepared according to literature procedure (Lee et al. 2011). To a solution of T315 (31.1 mg, 0.06 mmol) in THF (1 mL) was added propargyl bromide (7.9 mg, 0.066 mmol) followed by diisopropylethylamine (8.53 mg, 0.066 mmol). The reaction was left to stir at room temperature for 4 hours and then concentrated under vacuum. The resulting reside was purified by normal phase silica gel chromatography (EtOAc:CH_3CN:MeOH , 7:2.5:0.5, 0.1% Et_3N) to afford **2** (19 mg, 0.033 mmol, 55%) as an off-white solid. ^1H NMR (400 MHz, MeOD) δ 7.94 – 7.89 (m, 2H), 7.85 (d, J = 8.1 Hz, 2H), 7.76 – 7.71 (m, 4H), 7.40 – 7.34 (m, 2H), 7.14 – 7.08 (m, 2H), 6.69 (s, 1H), 3.39 (m, 2H), 3.35 – 3.32 (m, 4H), 2.94 (t, J = 7.5 Hz, 2H), 2.81 – 2.75 (m, 4H), 2.72 (t, J = 2.4 Hz, 1H), 2.70 (s, 3H), 2.52 (t, J = 7.5 Hz, 2H). ^{13}C NMR (101 MHz, MeOD) δ 174.71, 152.78, 152.04, 146.15, 140.18, 134.43, 132.15, 128.52, 128.38, 128.13, 127.38, 126.82, 126.78, 117.08, 103.63, 78.96, 75.28, 52.77, 35.50, 26.35, 23.13. HRMS m/z calc'd for $C_{33}H_{33}F_3N_5O$ $[M+H]^+$: 572.2637, found 572.2640.

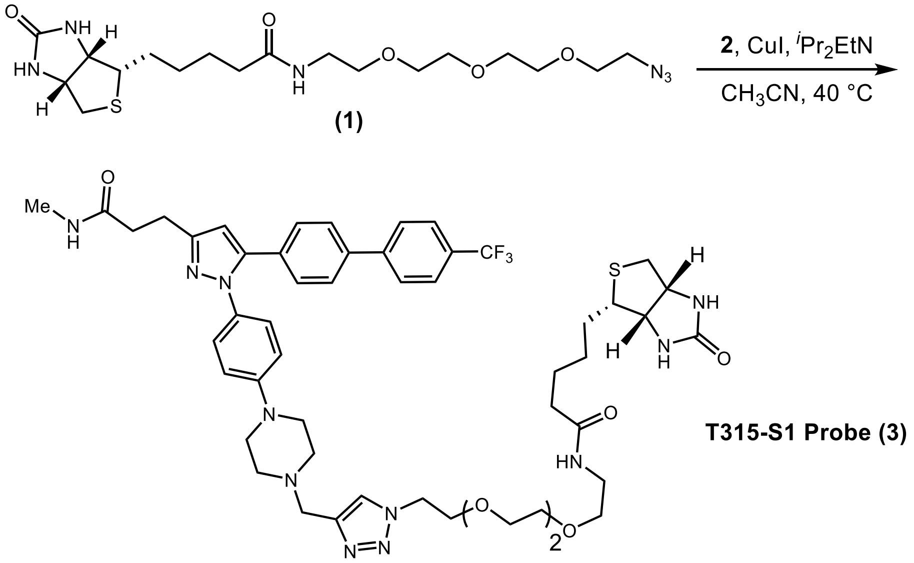

N-(2-(2-(2-(2-(4-((4-(4-(3-(3-(methylamino)-3-oxopropyl)-5-(4'-(trifluoromethyl)-[1,1'-biphenyl]-4-yl)-1H-pyrazol-1-yl)phenyl)piperazin-1-yl)methyl)-1H-1,2,3-triazol-1-yl)ethoxy)ethoxy)ethoxy)ethyl)-5-((3aS,4S,6aR)-2-oxohexahydro-1H-thieno[3,4-d]imidazol-4-yl)pentanamide. Azide biotin linker **1** (8.4 mg, 0.02 mmol) was added to a solution of T315 alkyne **2**, (10.9 mg, 0.02 mmol) in CH_3CN (1 mL), followed by copper iodide (0.4 mg, 0.0022 mmol) and diisopropylethylamine (2.8 mg, 0.022 mmol). The reaction was left to stir at 40 °C for 12 hours under nitrogen. Upon completion, the reaction was filtered over a pad of Celite and concentrated. The residue was taken up in a small amount of acetonitrile and purified by normal phase silica gel chromatography (EtOAc:CH_3CN:MeOH, 3:6.5:0.5, 0.2% Et_3N). Pooled fractions were collected and concentrated under vacuum to afford **T315-S1 Probe, 3** as a clear film (11.3 mg, 0.01 mmol, 56%). ^1H NMR (300 MHz, MeOD) δ 8.76 (s, 1H), 7.96 – 7.84 (m, 5H), 7.78 – 7.71 (m, 4H), 7.43 (m, 2H), 7.18 (d, J = 8.7 Hz, 2H), 6.71 (s, 1H), 3.99 (t, J = 6.6 Hz, 2H), 3.64 (m, 6H), 3.54 (m, 2H), 3.50 – 3.35 (m, 11H), 3.18 (m, 3H), 3.07 (m, 1H), 2.98 – 2.93 (m, 2H), 2.70 (s, 3H), 2.56 – 2.50 (m, 2H), 1.64 (m, 10H), 1.14 – 1.07 (m, 4H). HRMS m/z calc'd for $C_{51}H_{64}F_3N_{11}NaO_6S$ $[M+Na]^+$: 1038.4612, found 1038.4670.

Part III: Synthesis of T315-S2 Probe

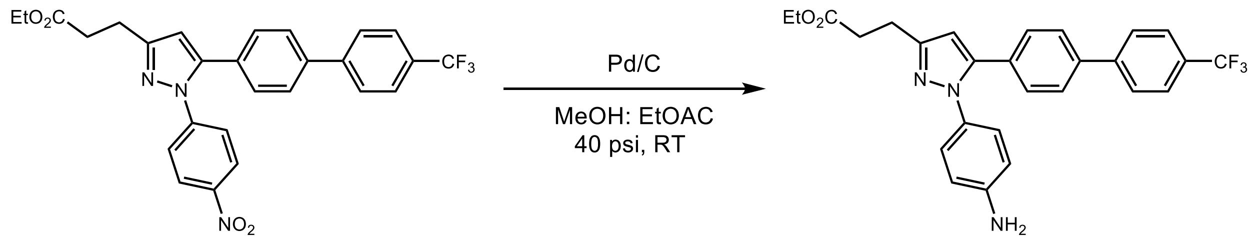

(4)

Ethyl 3-(1-(4-aminophenyl)-5-(4'-(trifluoromethyl)-[1,1'-biphenyl]-4-yl)-1H-pyrazol-3-yl)propanoate. Synthesis of T-315 S2 (**8**) began from a known intermediate ethyl 3-(1-(4-nitrophenyl)-5-(4'-(trifluoromethyl)-[1,1'-biphenyl]-4-yl)-1H-pyrazol-3-yl)propanoate (**2**) that was prepared following literature procedure.[2] To a solution of ethyl 3-(1-(4-nitrophenyl)-5-(4'-(trifluoromethyl)-[1,1'-biphenyl]-4-yl)-1H-pyrazol-3-yl)propanoate (464.4 mg, 0.91 mmol) in MeOH:EtOAc (1:3, 10 mL) was added Pd/C (14 mg). The reaction was stirred under H_2 (40 psi, room temperature) for 1 hour. The reaction mixture was filtered over celite and concentrated to afford ethyl 3-(1-(4-aminophenyl)-5-(4'-(trifluoromethyl)-[1,1'-biphenyl]-4-yl)-1H-pyrazol-3-yl)propanoate, **4** (426.1 mg, 0.89 mmol, 98%) as a brown foam that was carried forward without further purification. ^{1}H NMR (400 MHz, $CDCl_3$) δ 7.96 (d, J = 8.5 Hz, 2H), 7.73 (m, 4H), 7.66 (d, J = 8.5 Hz, 2H), 7.27 (d, J = 8.0 Hz, 2H), 6.77 (d, J = 8.7 Hz, 2H), 6.56 (s, 1H), 4.16 (q, J = 7.1 Hz, 2H), 2.99 (t, J = 7.6 Hz, 2H), 2.62 (t, J = 7.6 Hz, 2H), 1.23 (t, J = 7.1 Hz, 3H). ^{13}C NMR (176 MHz, $CDCl_3$) δ 172.14, 150.84, 144.32, 144.02, 139.17, 127.58, 127.38, 127.30, 127.10, 126.40, 125.85, 125.83, 102.92, 60.93, 33.19, 21.79, 14.34. HRMS m/z calc'd for $C_{27}H_{25}F_3N_3O_2$ $[M+H]^+$: 480.1899, found 480.1869.

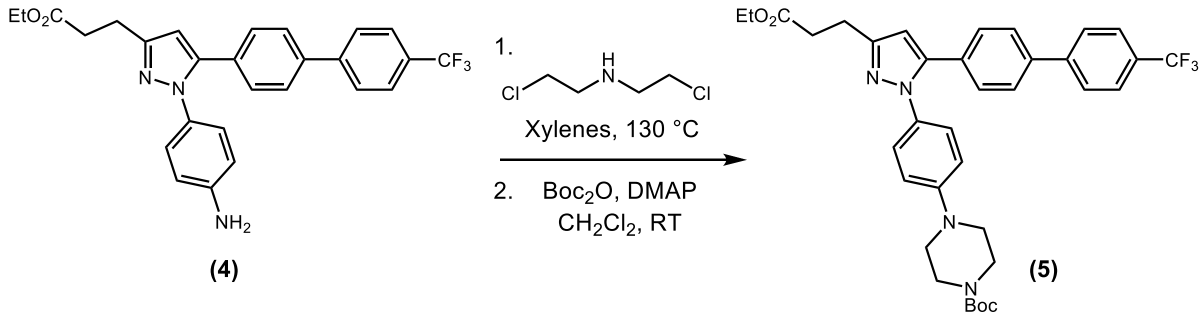

tert-Butyl 4-(4-(3-(3-ethoxy-3-oxopropyl)-5-(4'-(trifluoromethyl)-[1,1'-biphenyl]-4-yl)-1H-pyrazol-1-yl)phenyl)piperazine-1-carboxylate. To a flask containing a solution of **4** (517.9 mg, 1.08 mmol) in xylenes (11 mL) was added bis(2-chloroethyl)amine (184.1 mg, 1.30 mmol). After heating at 130 °C for 72 hours, the reaction was cooled to room temperature and concentrated under vacuum to afford ethyl 3-(1-(4-(piperazin-1-yl)phenyl)-5-(4'-(trifluoromethyl)-[1,1'-biphenyl]-4-yl)-1H-pyrazol-3-yl)propanoate HRMS m/z calc'd for $C_{31}H_{32}F_3N_4O_2$ [M+H]+: 549.2477, found 549.5000.

The resulting residue (75.5 mg, 0.14 mmol) was taken up in methylene chloride (13.8 mL) followed by the addition of di-*tert*-butyl dicarbonate (31.5 mg, 0.144 mmol), then 4-dimethylaminopyridine (1.67 mg, 0.014 mmol). The reaction was left to stir at room temperature for 3 hours and then concentrated under vacuum. The residue was purified by normal phase silica gel chromatography (1% - 5% MeOH in CH_2Cl_2, 0.1% Et_3N) to afford **5** as a colorless foam (47.4 mg, 0.073 mmol, 53%). ^1H NMR (400 MHz, $CDCl_3$) δ 7.94 (d, J = 8.4 Hz, 2H), 7.71 (m, 4H), 7.63 (d, J = 8.4 Hz, 2H), 7.39 (d, J = 8.8 Hz, 2H), 7.03 (d, J = 8.6 Hz, 2H), 6.55 (s, 1H), 4.14 (q, J = 7.1 Hz, 2H), 3.67 – 3.58 (m, 4H), 3.26 – 3.17 (m, 4H), 2.99 (t, J = 7.6 Hz, 2H), 2.65 (t, J = 7.6 Hz, 2H), 1.50 (s, 9H), 1.24 (t, J = 7.1 Hz, 3H). ^{13}C NMR (101 MHz, $CDCl_3$) δ 172.25, 154.79, 143.70, 138.92, 133.42, 127.53, 127.31, 126.81, 126.33, 125.84, 125.80, 102.58, 80.21, 60.86, 33.33, 28.56, 21.80, 14.33. HRMS m/z calc'd for $C_{36}H_{40}F_3N_4O_4$ $[M+H]^+$: 649.3002, found 649.2995.

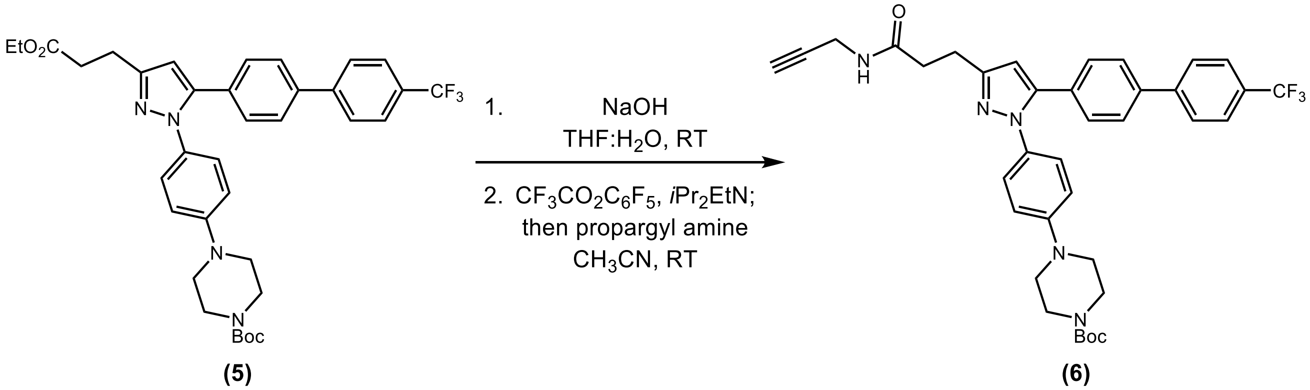

***tert*-Butyl 4-(4-(3-(3-oxo-3-(prop-2-yn-1-ylamino)propyl)-5-(4'-(trifluoromethyl)-[1,1'-biphenyl]-4-yl)-1H-pyrazol-1-yl)phenyl)piperazine-1-carboxylate.** To a solution of **5** (25.1 mg, 0.039 mmol) in a solution of THF:H_2O (3:1, 1 mL) was added 3 M NaOH (4.64 mg, 0.116 mmol). The reaction was left to stir at room temperature for 3 hours. TLC indicated complete hydrolysis of ester. The reaction was concentrated to remove THF. Water (3mL) was added and the solution was extracted with ethyl acetate (4 ml x 3), dried over sodium sulfate, filtered and concentrated to afford 3-(1-(4-(4-(tert-butoxycarbonyl)piperazin-1-yl)phenyl)-5-(4'-(trifluoromethyl)-[1,1'-biphenyl]-4-yl)-1H-pyrazol-3-yl)propanoic acid. The residue was immediately dissolved in anhydrous acetonitrile followed by the addition of pentafluorophenyl trifluoroacetate (12.01 mg, 0.043 mmol) and diisopropylethylamine (5.56 mg, 0.043mmol). The reaction was allowed to stir at room temperature for 2 hours, followed by the dropwise addition of propargyl amine (2.37 mg, 0.043 mmol). After 2 hours, the reaction was concentrated and purified by normal phase chromatography (0.5% - 1% MeOH in CH_2Cl_2, 0.1% Et_3N) to afford **6** (20.2 mg, 0.031 mmol, 78%). as a colorless foam. ^{1}H NMR (300 MHz, $CDCl_3$) δ 7.95 – 7.89 (m, 2H), 7.66 (m, 7H), 7.41 (d, J = 8.9 Hz, 2H), 7.13 (d, J = 7.5 Hz, 2H), 6.56 (s, 1H), 4.03 (dd, J = 5.2, 2.5 Hz, 2H), 3.73 – 3.61 (m, 4H), 3.28 – 3.20 (m, 4H), 3.06 – 2.98 (m, 2H), 2.56 – 2.46 (m, 2H), 2.21 (m, 1H), 1.50 (s, 9H). ^{13}C NMR (75 MHz, $CDCl_3$) δ 170.65, 154.57, 150.78, 144.23, 143.79, 138.99, 133.01, 127.43, 127.18, 126.72, 126.26, 126.10, 125.73, 125.68, 102.83, 80.35, 79.32, 77.44, 77.22, 77.02, 76.59, 71.76, 34.93, 29.27, 28.41, 21.89. HRMS m/z calc'd for $C_{37}H_{39}F_3N_5O_3$ $[M+H]^+$: 658.3000, found 658.3014.

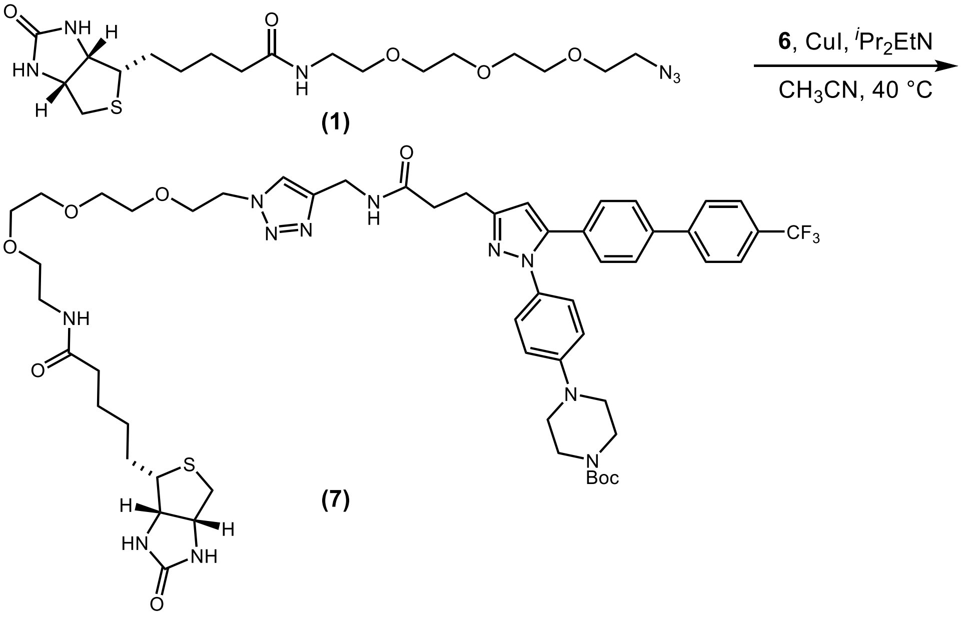

***tert*-Butyl 4-(4-(3-(3-oxo-3-(((1-(13-oxo-17-((3aS,4S,6aR)-2-oxohexahydro-1H-thieno[3,4-d]imidazol-4-yl)-3,6,9-trioxa-12-azaheptadecyl)-1H-1,2,3-triazol-4-yl)methyl)amino)propyl)-5-(4'-(trifluoromethyl)-[1,1'-biphenyl]-4-yl)-1H-pyrazol-1-yl)phenyl)piperazine-1-carboxylate).** To a solution of **6** (11.3 mg, 0.017 mmol) in CH_3CN (1 mL) stirring under argon was added biotin

azide **1** (7.6 mg, 0.017 mmol), copper iodide (0.36 mg, 0.0019 mmol), followed by diisopropylethylamine (2.4 mg, 0.019 mmol. The reaction was left to stir at 40 °C for 2 hours then filtered over celite and concentrated under vacuum. The residue was purified by normal phase silica gel chromatography (EtOAc:CH_3CN:MeOH, 5:4:1, 0.1% Et_3N) to afford **7** (13.3 mg, 0.012 mmol, 71%) as a light brown film. ^{1}H NMR (400 MHz, MeOD) δ 7.89 (s, 1H), 7.82 (d, J = 8.2 Hz, 2H), 7.73 (d, J = 8.4 Hz, 2H), 7.65 (d, J = 8.5 Hz, 2H), 7.33 (d, J = 8.4 Hz, 2H), 7.15 (d, J = 9.0 Hz, 2H), 6.99 (d, J = 9.0 Hz, 2H), 6.46 (s, 1H), 4.48 – 4.44 (m, 4H), 4.27 (m, 1H), 3.84 – 3.79 (m, 2H), 3.57 (m, 10H), 3.50 (m, 2H), 3.20 (m, 6H), 3.04 (t, J = 7.2 Hz, 2H), 2.91 (m, 1H), 2.67 (m, 3H), 2.18 (m, 2H), 1.74 – 1.52 (m, 8H), 1.48 (s, 9H), 1.42 (m, 4H), 1.11 (t, J = 7.3 Hz, 2H). HRMS m/z calc'd for $C_{55}H_{70}F_3N_{11}NaO_8S$ $[M+Na]^+$: 1124.4979, found 1124.4975.

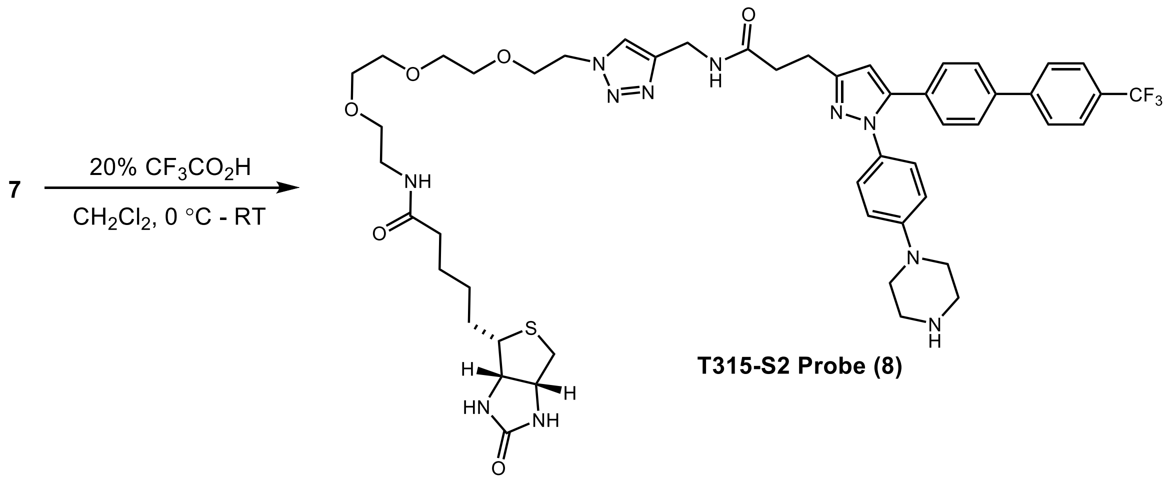

5-((3aS,4S,6aR)-2-oxohexahydro-1H-thieno[3,4-d]imidazol-4-yl)-N-(2-(2-(2-(2-(4-((3-(1-(4-(piperazin-1-yl)phenyl)-5-(4'-(trifluoromethyl)-[1,1'-biphenyl]-4-yl)-1H-pyrazol-3-yl)propanamido)methyl)-1H-1,2,3-triazol-1-yl)ethoxy)ethoxy)ethoxy)ethyl)pentanamide. To a vial containing **7** (13 mg, 0.012 mmol) was added a 20% solution of trifluoroacetic acid in methylene chloride (0.5 mL) slowly at 0 °C. The reaction was allowed to gradually warm up to room temperature and left to stir for 3 hours. The reaction was then concentrated to dryness and the resulting residue was taken up in a minimal amount of methylene chloride and purified by normal phase silica gel chromatography (EtOAc: CH_3CN:MeOH, 3:6.5:0.5, 0.2% Et_3N) to afford **T315-S2 Probe, 8** (6.6 mg, 0.007 mmol 55%), as an off-white film. ^{1}H NMR (400 MHz, MeOD) δ 7.88 (s, 1H), 7.81 (d, J = 8.2 Hz, 2H), 7.73 (d, J = 8.4 Hz, 2H), 7.64 (d, J = 8.2 Hz, 2H), 7.33 (d, J = 8.3 Hz, 2H), 7.19 (d, J = 8.9 Hz, 1H), 7.16 – 7.11 (m, 1H), 7.03 (d, J = 8.9 Hz, 1H), 7.01 – 6.94 (m, 1H), 6.45 (s, 1H), 4.50 – 4.42 (m, 5H), 4.27 (m, 1H), 3.81 (m, 2H), 3.56 (m, 7H), 3.52 – 3.47 (m, 3H), 3.44 – 3.40 (m, 2H), 3.19 (m, 3H), 3.03 (t, J = 7.5 Hz, 2H), 2.89 (m, 1H), 2.66 (m, 3H), 2.18 (t, J = 7.3 Hz, 2H), 1.76 – 1.35 (m, 10H), 1.17 (t, J = 7.0 Hz, 2H), 1.10 (t, J = 7.3 Hz, 2H). HRMS m/z calc'd for $C_{50}H_{62}F_3N_{11}NaO_6S$ $[M+Na]^+$: 1024.4455, found 1024.4470.

References:

Chambers, J. M., L. M. Lindqvist, A. Webb, D. C. Huang, G. P. Savage, and M. A. Rizzacasa. 2013. "Synthesis of biotinylated episilvestrol: highly selective targeting of the translation factors eIF4AI/II." *Org Lett* 15 (6):1406-9. doi: 10.1021/ol400401d.

Lee, S. L., E. C. Hsu, C. C. Chou, H. C. Chuang, L. Y. Bai, S. K. Kulp, and C. S. Chen. 2011. "Identification and characterization of a novel integrin-linked kinase inhibitor." *J Med Chem* 54 (18):6364-74. doi: 10.1021/jm2007744.

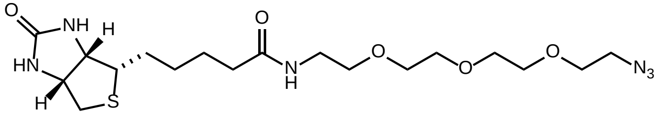

azide biotin linker, 1

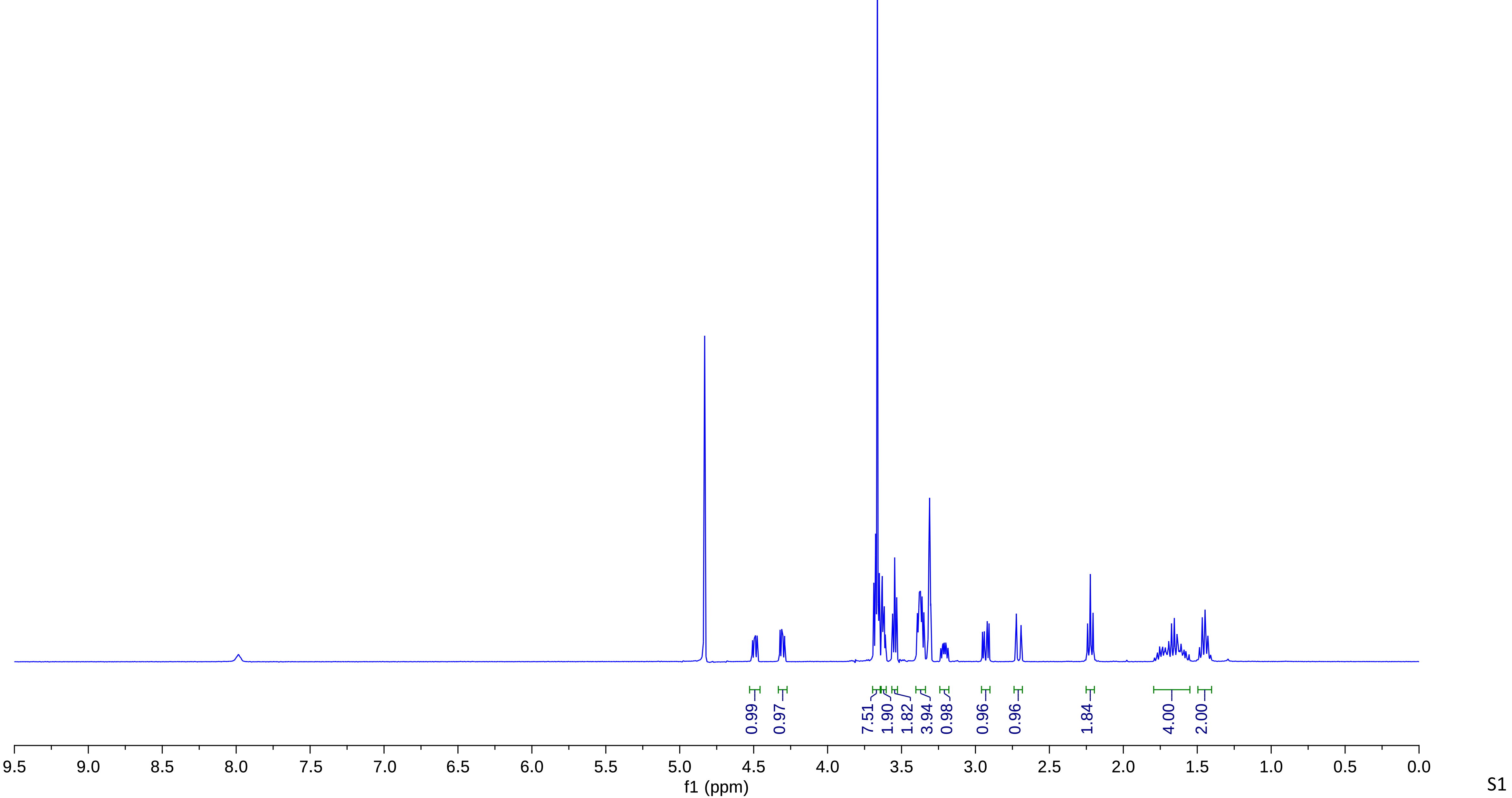

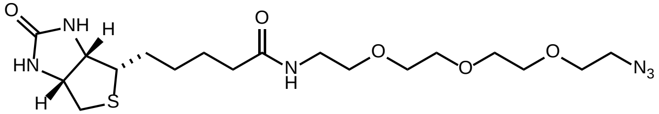

azide biotin linker, 1

^{13}C NMR (101 MHz, MeOD)

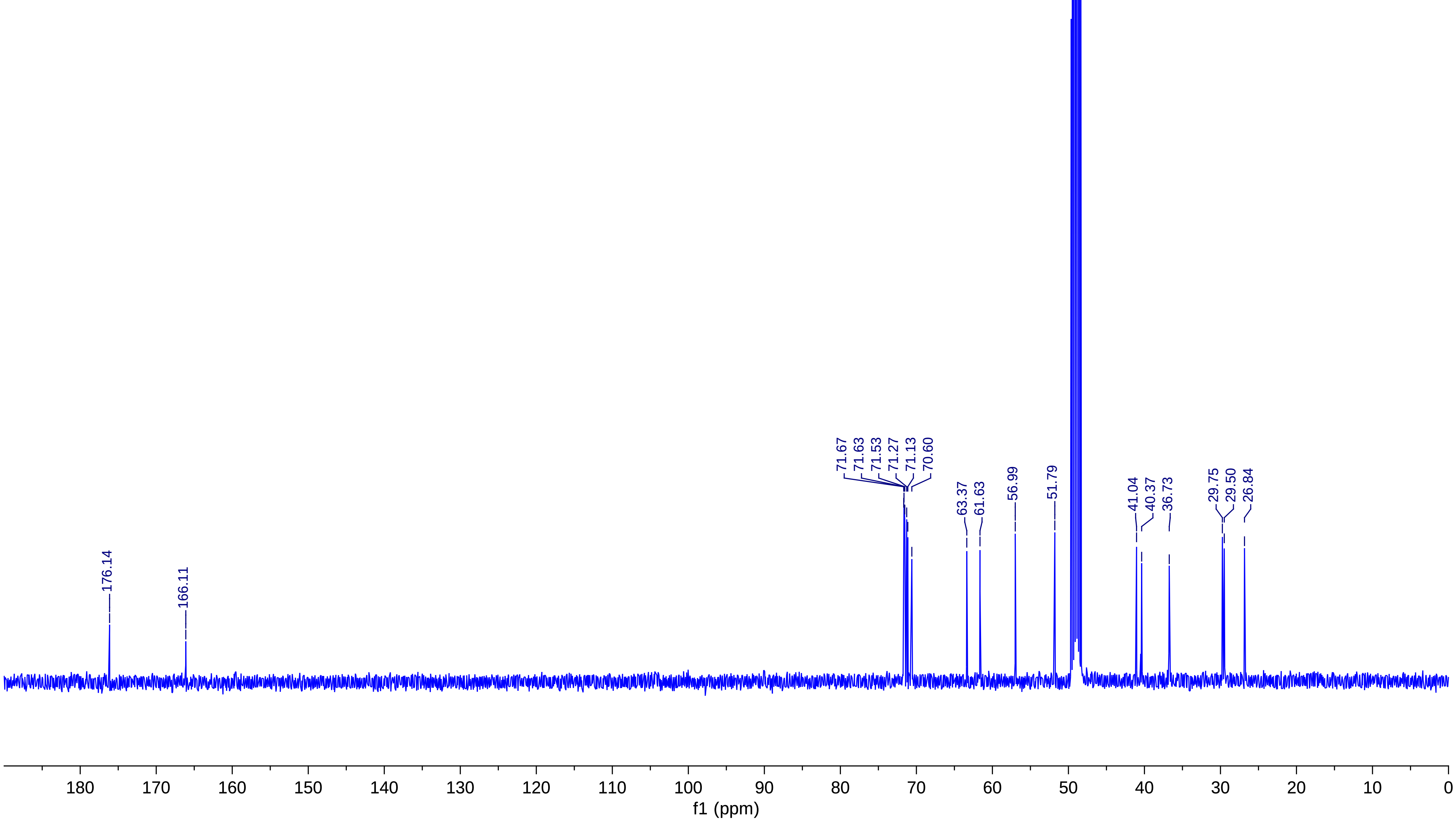

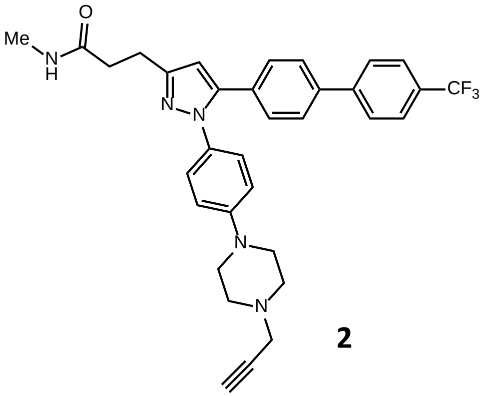

2

^{1}H NMR (400 MHz, MeOD)

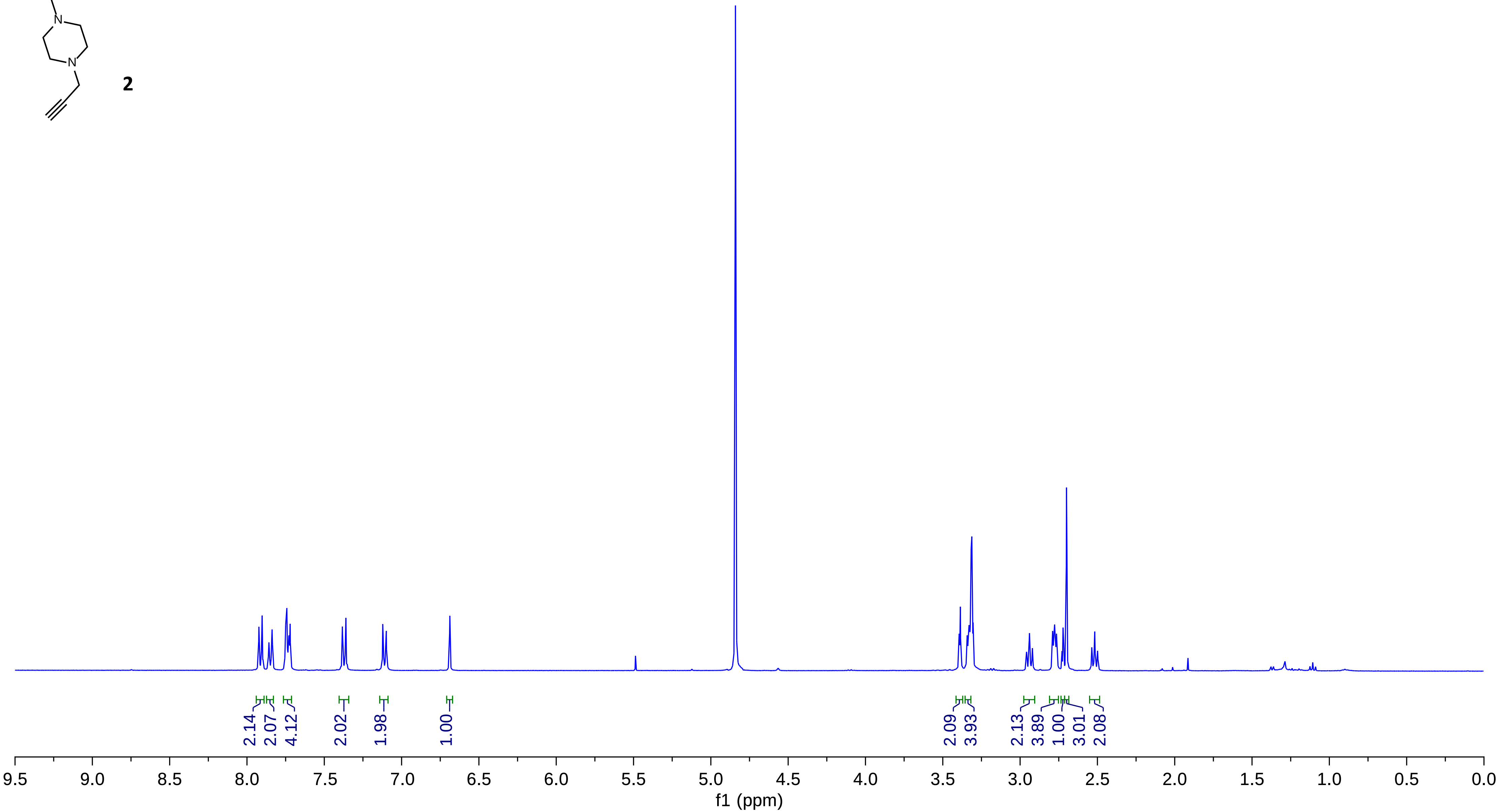

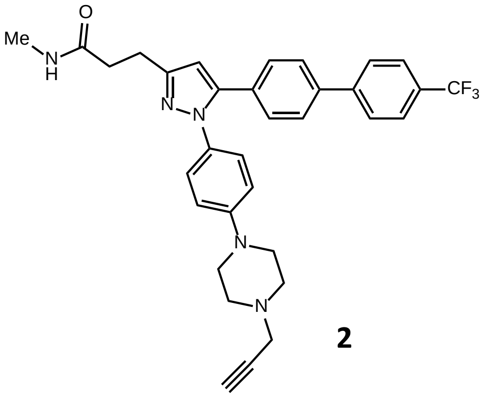

2

^{13}C NMR (101 MHz, MeOD)

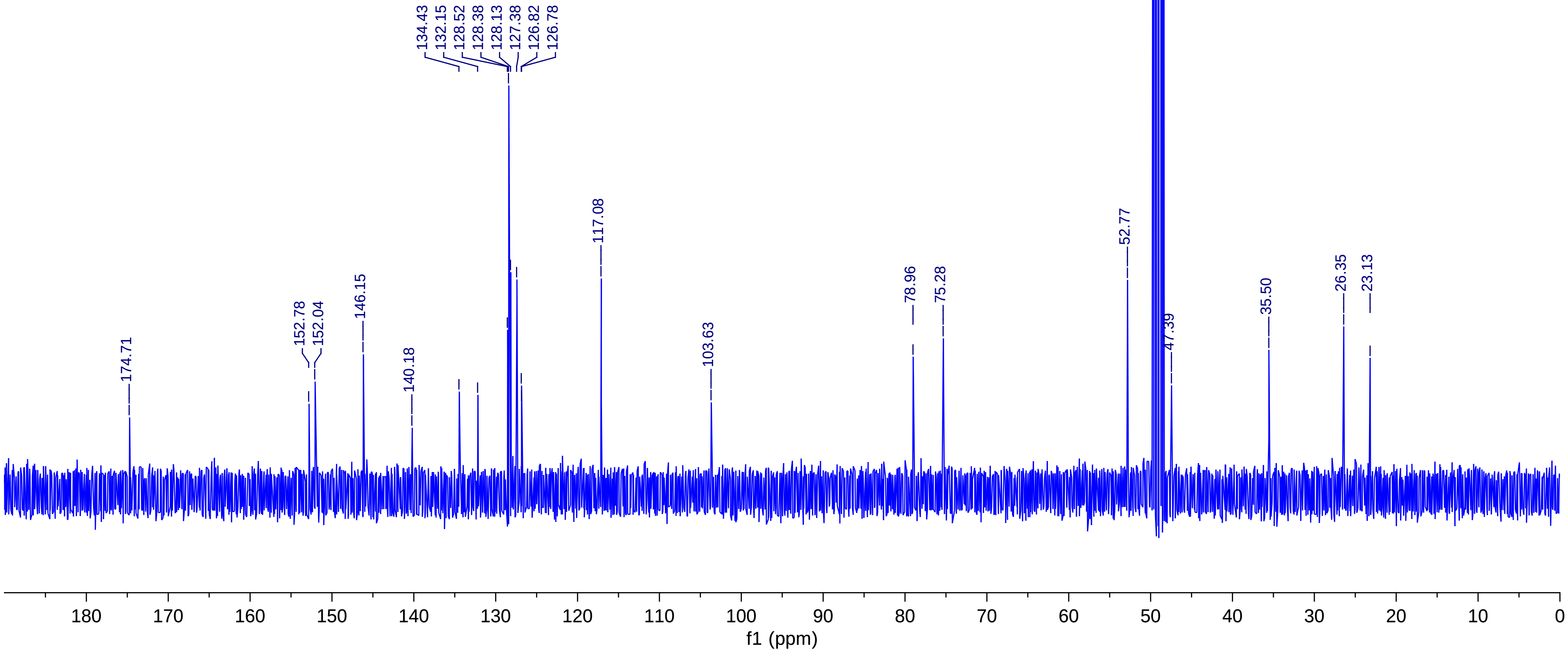

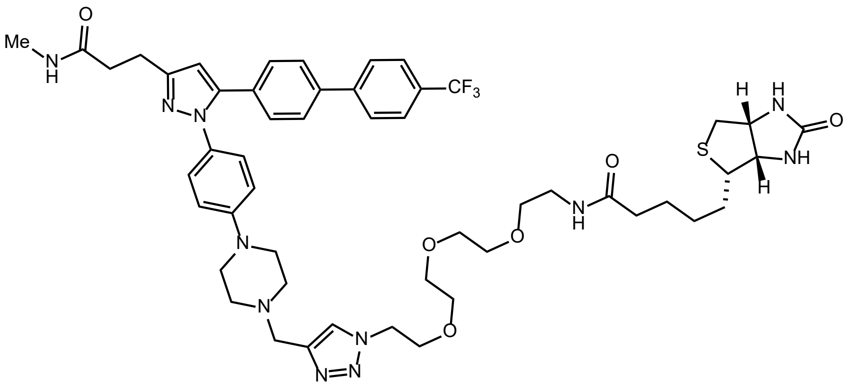

T315-S1 Probe, 3

^{1}H NMR (300 MHz, MeOD)

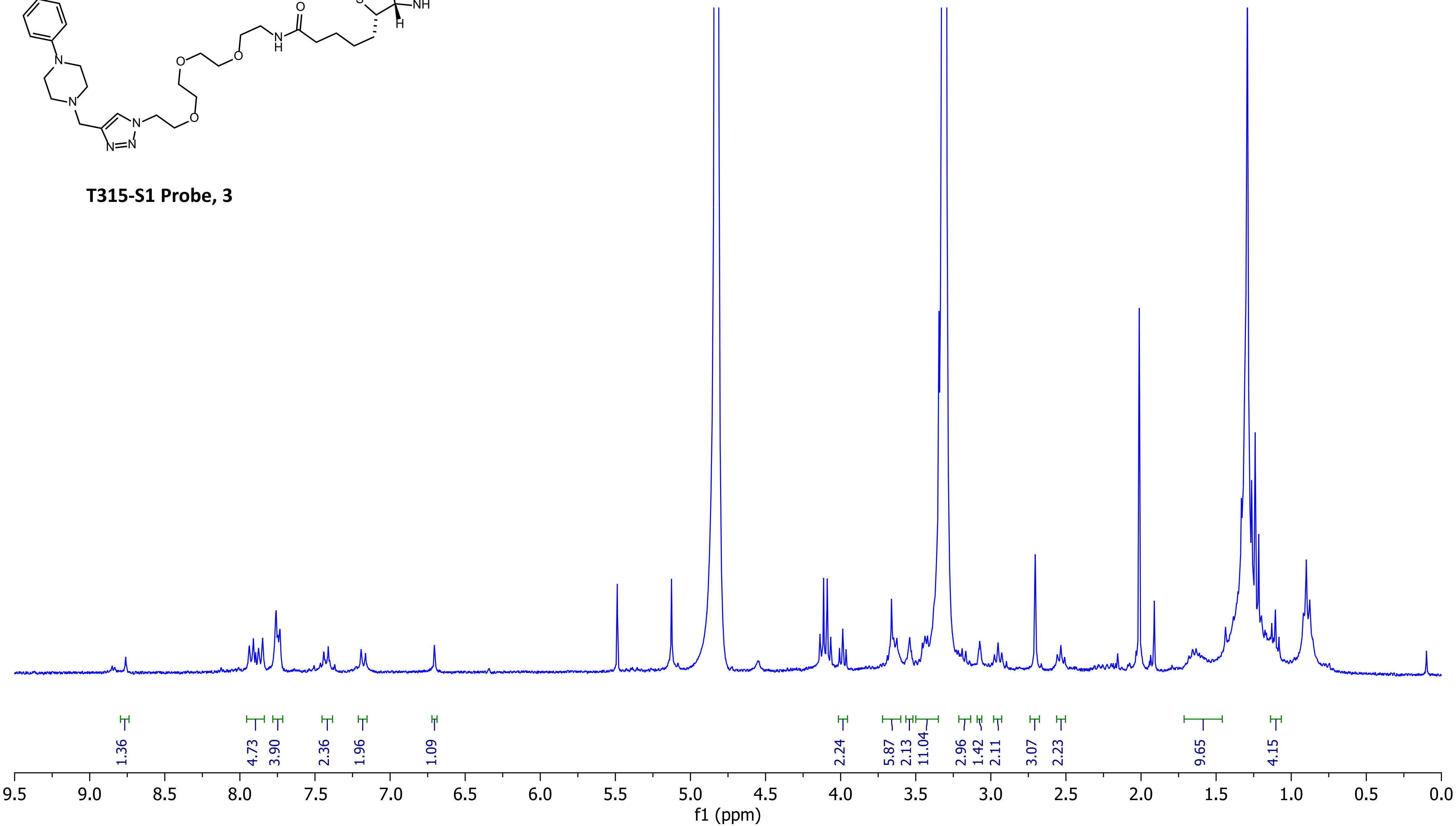

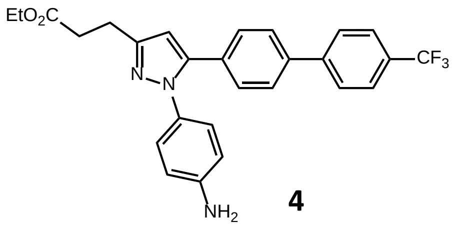

4

^{1}H NMR (400 MHz, $CDCl_3$)

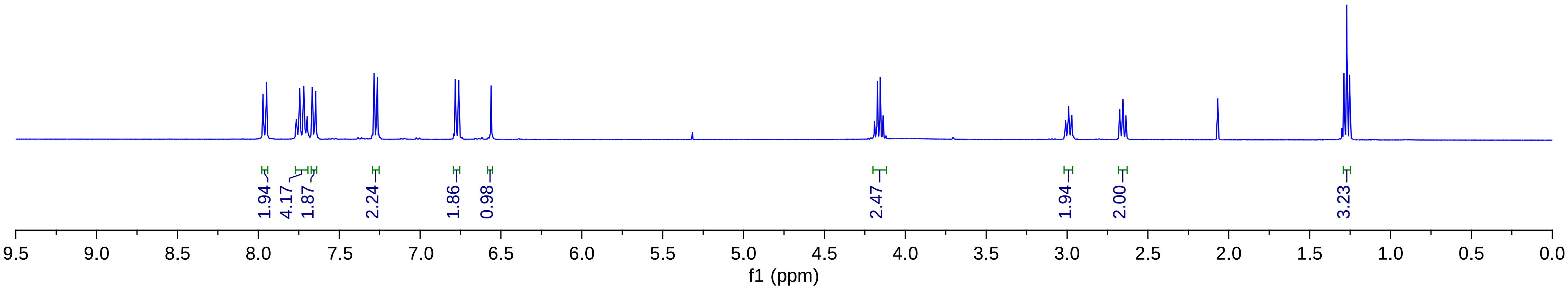

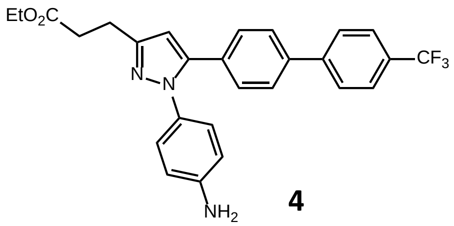

4

^{13}C NMR (176 MHz, $CDCl_3$)

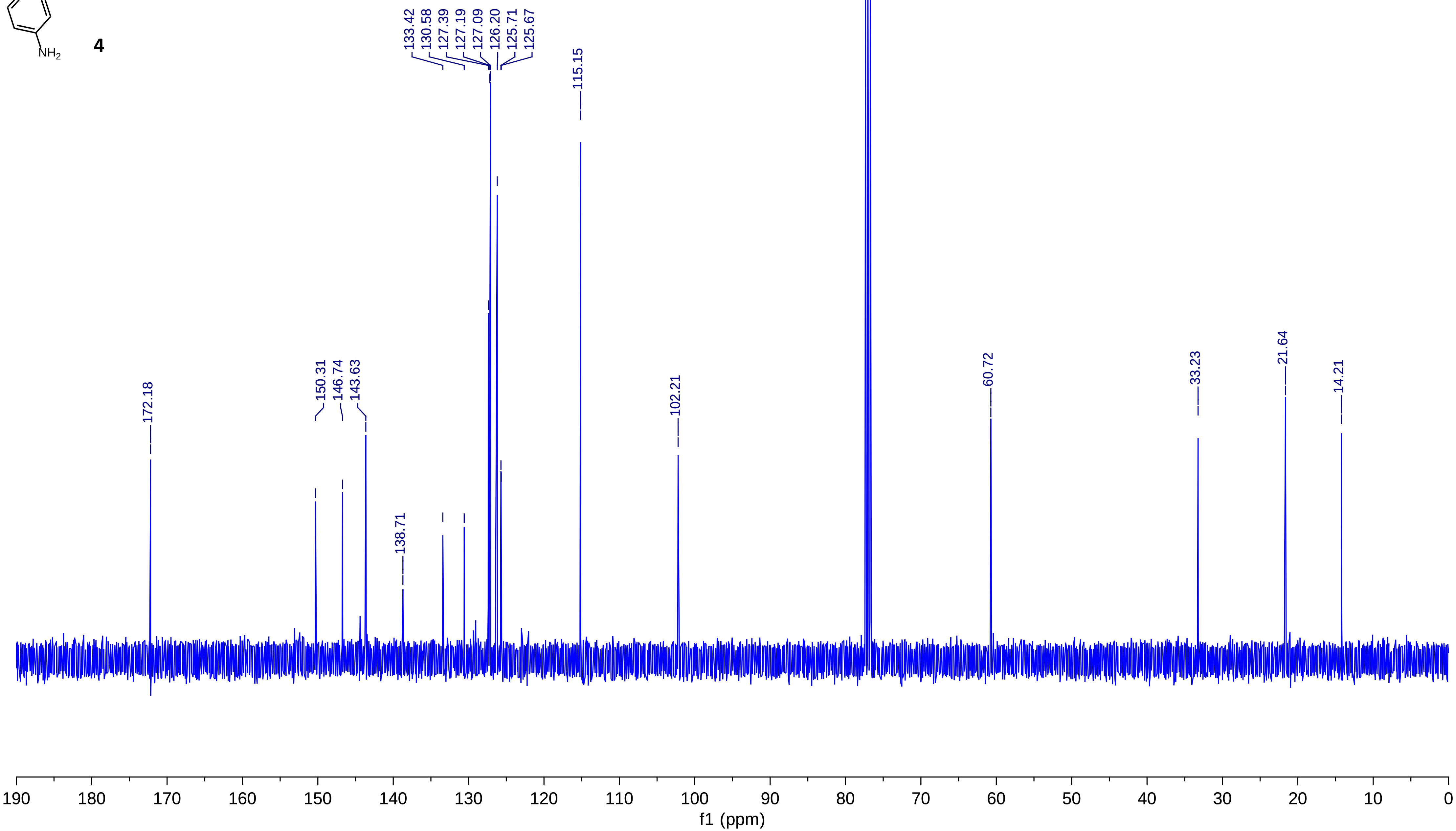

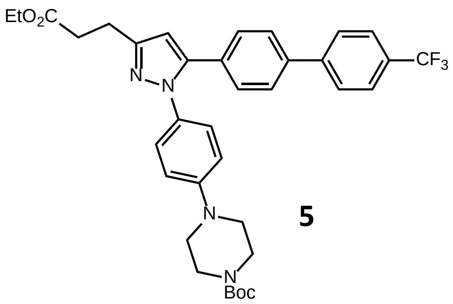

5

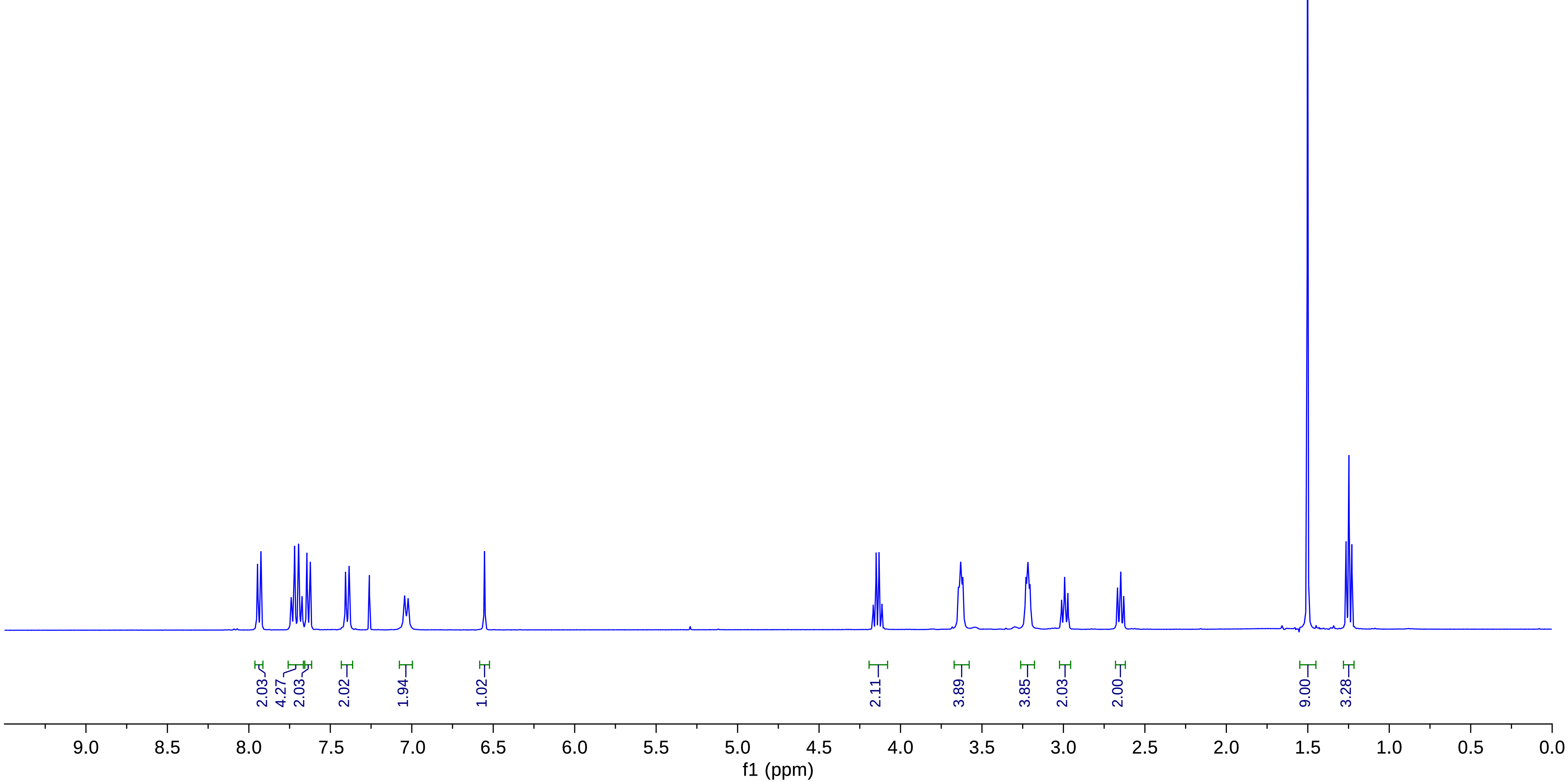

^1H NMR (400 MHz, $CDCl_3$)
2.03
4.27
2.03
2.02
1.94
1.02
2.11
3.89
3.85
2.03
2.00
9.00
3.28
9.0
8.5
8.0
7.5
7.0
6.5
6.0
5.5
5.0
4.5
4.0
3.5
3.0
2.5
2.0
1.5
1.0
0.5
0.0
f1 (ppm)

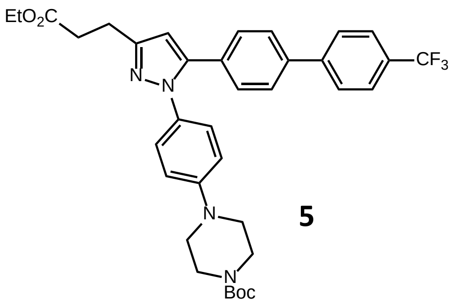

5

^{13}C NMR (101 MHz, $CDCl_3$)

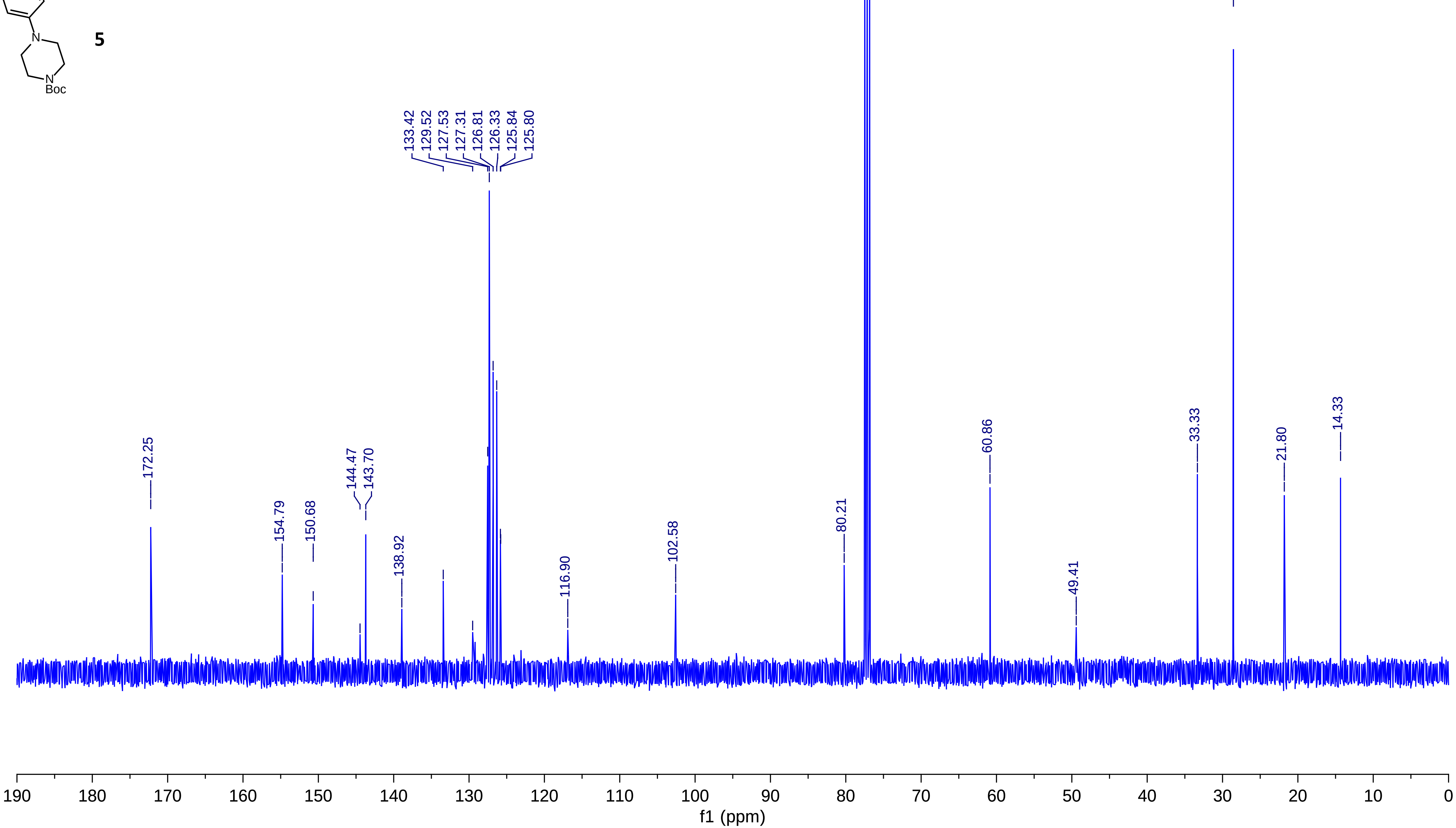

^1H NMR (300 MHz, $CDCl_3$)

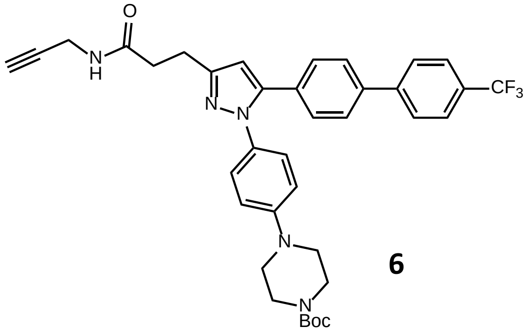

6

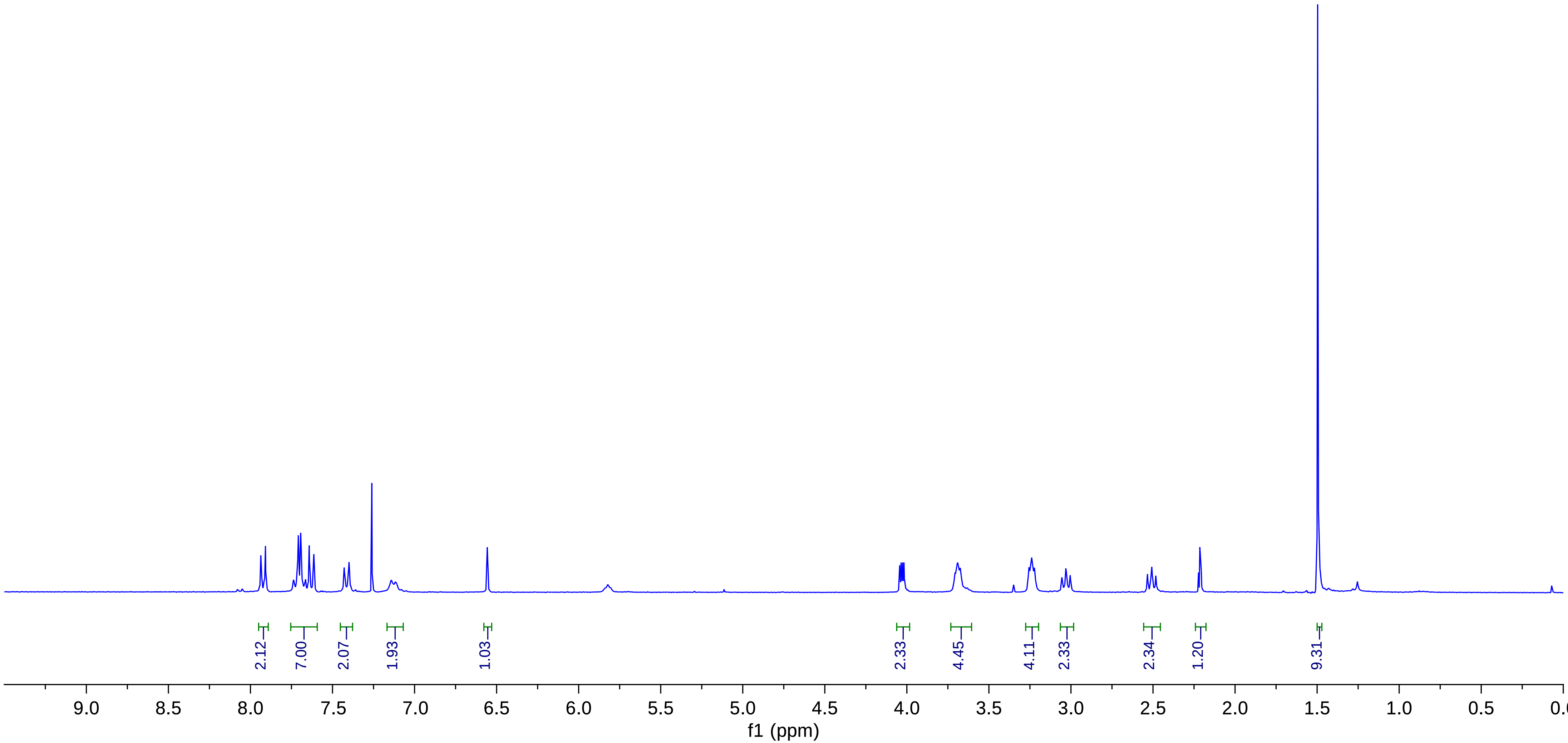

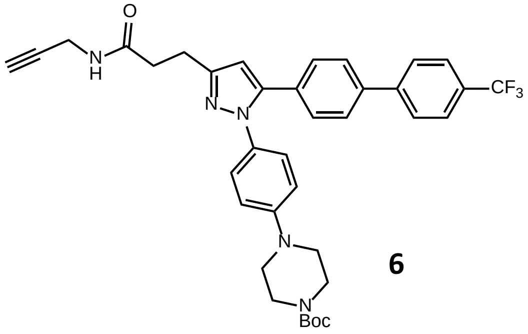

6

^{13}C NMR (75 MHz, $CDCl_3$)

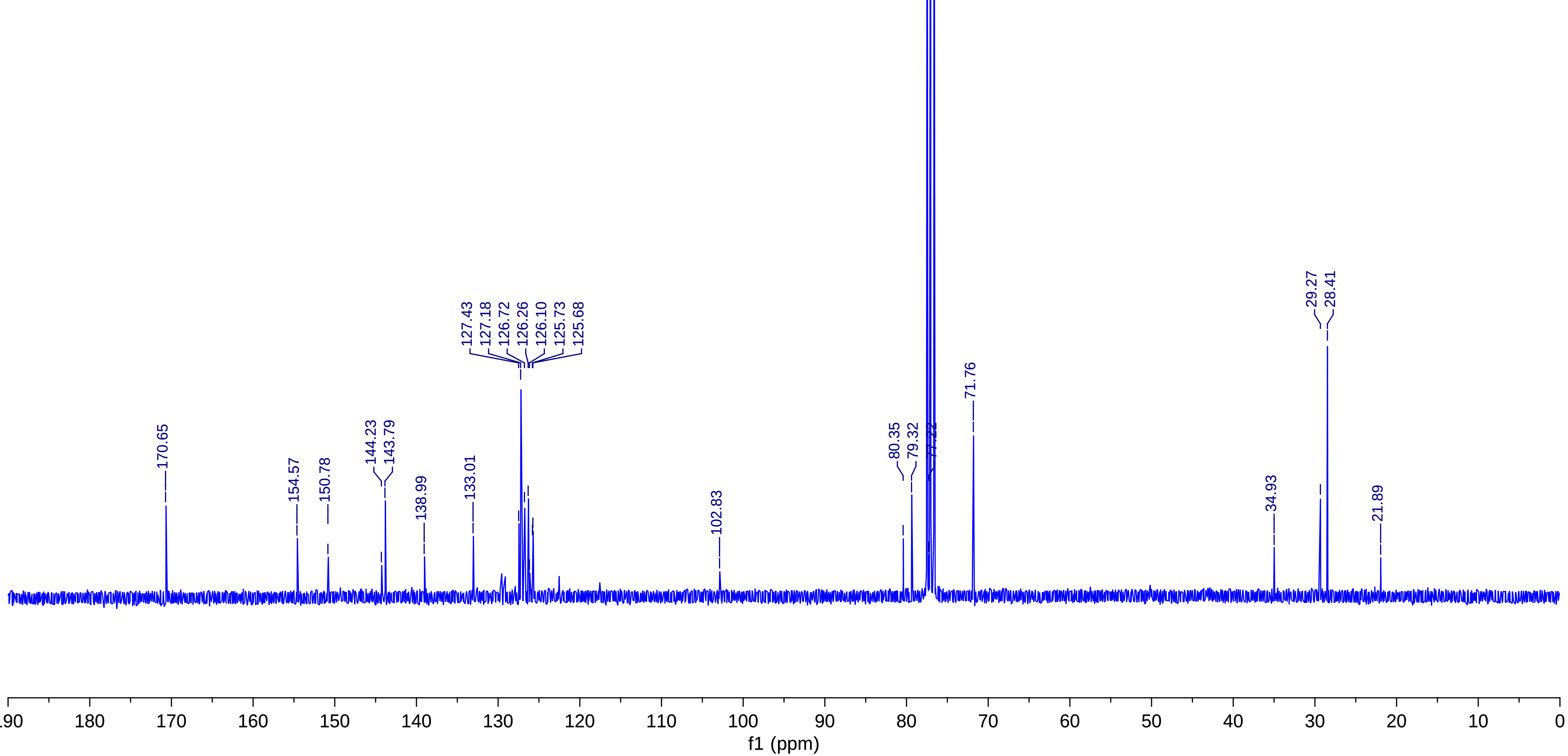

^{1}H NMR (400 MHz, MeOD)

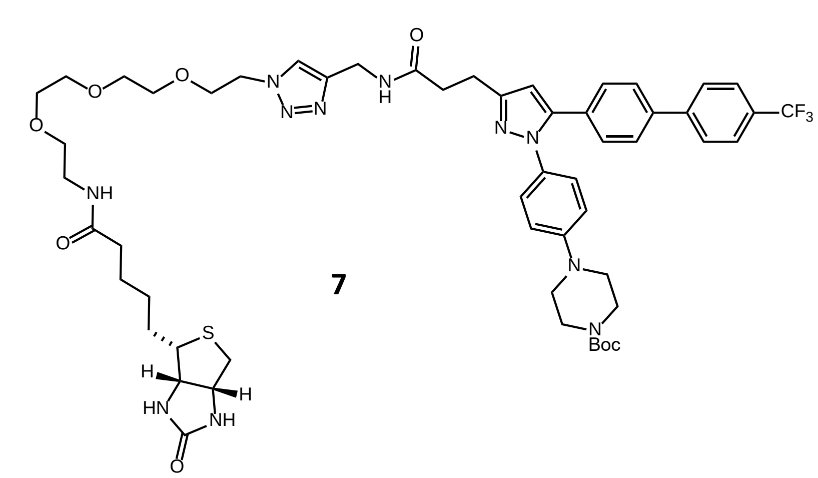

7

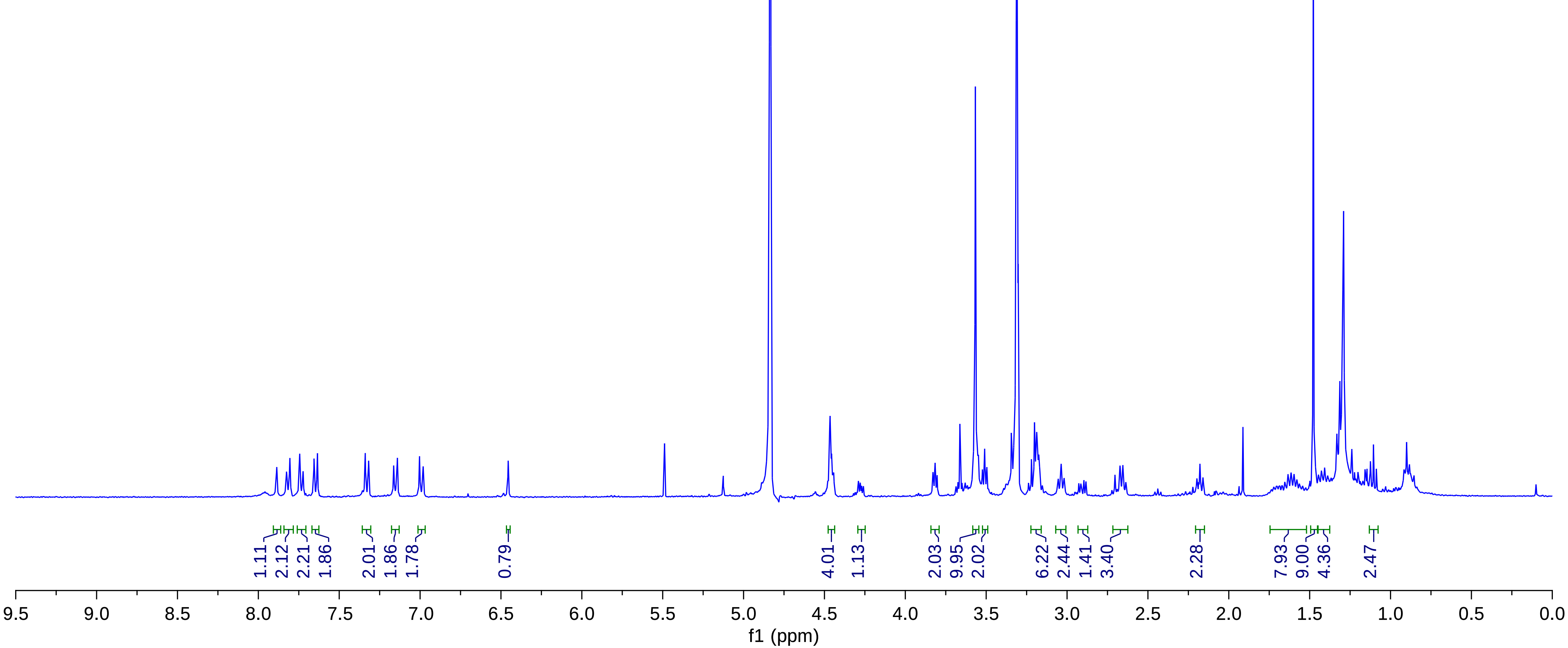

^1H NMR (400 MHz, MeOD)

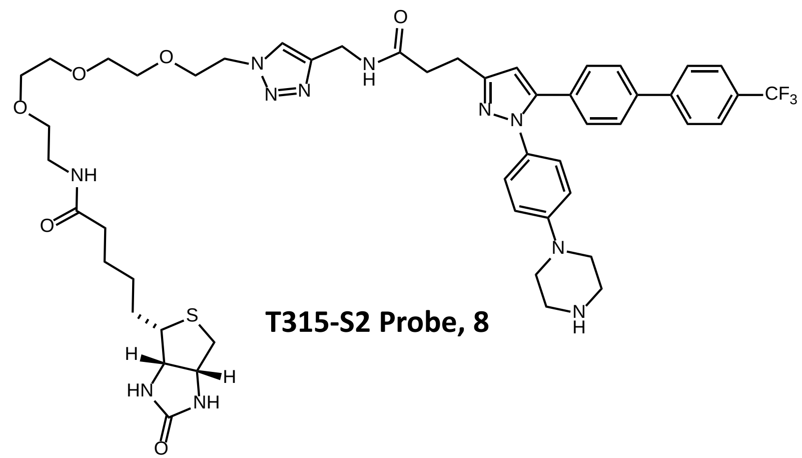

T315-S2 Probe, 8

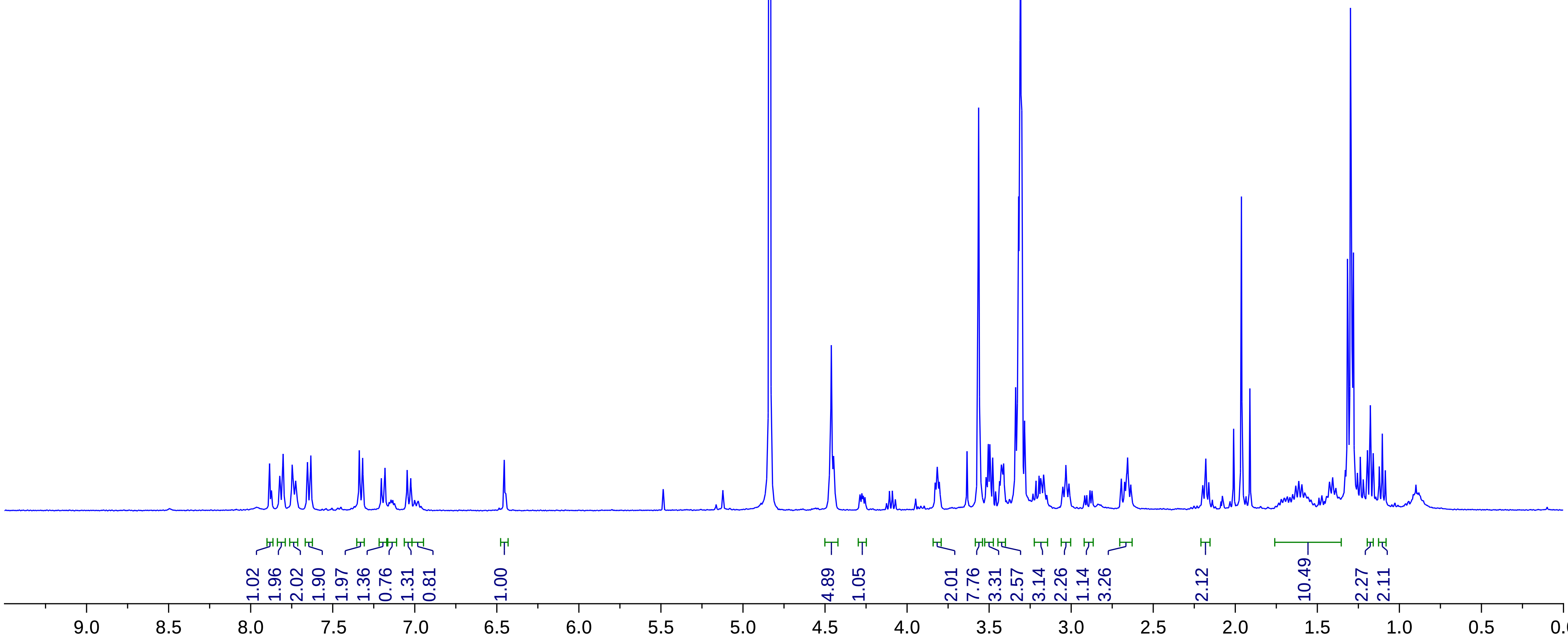

Supplement: Supplementary file 1 [file Data_Sheet_1.pdf]
